# Women’s health is a team effort: probiogenomics supports the development of a multi-species vaginal probiotic

**DOI:** 10.1007/s00018-026-06107-2

**Published:** 2026-02-26

**Authors:** Chiara Maria Calvanese, Vincenzo Valentino, Annachiara De Prisco, Serena Allesina, Angela Amoruso, Francesca Deidda, Annalisa Visciglia, Danilo Ercolini, Marco Pane, Francesca De Filippis

**Affiliations:** 1 Department of Agricultural Sciences, University of Naples Federico II, P.zza Carlo di Borbone 1, Portici (NA), 80055 Italy; 2 Probiotical Research S.r.l, via Enrico Mattei 3, Novara, 28100 Italy; 3 Task Force on Microbiome Studies, University of Naples Federico II, Corso Umberto I 43, Napoli, 80100 Italy

**Keywords:** Vaginal microbiome, Vaginal fitness, Oral probiotic, Vaginal probiotic, Pangenome analysis.

## Abstract

The healthy vaginal microbiota is typically dominated by *Lactobacillus* species, while the dominance of different taxa often signals dysbiosis. Vaginal probiotics offer a promising therapeutic avenue to restore microbial balance and prevent recurrent infections. Using probiogenomics, this study investigated 19 novel strains belonging to four *Lactobacillus* species (*Lactobacillus crispatus*,* Lactobacillus gasseri*,* Lactobacillus paragasseri*,* Limosilactobacillus fermentum*) isolated from the vaginal environment of fertile and menopausal women. Through genomic screening and comparative genomics, we identified complementary roles between these species in activities crucial to women’s health. Our results indicate that vaginal *Lactobacillus* strains are genomically adapted to this niche, promoting persistence and pathogen-fighting. Furthermore, we demonstrated the potential ability of vaginal lactobacilli to survive the gastrointestinal transit and to explicate beneficial activities at the intestinal level, suggestion the possibility to be used through oral supplementation when topical application is not feasible. Although we confirmed *L. crispatus* as the most specialized to the vaginal niche, *L. gasseri* showed equivalent and complementary genetic traits, highlighting its previously underestimated role. *L. paragasseri* showed an interesting dichotomy, with beneficial traits alongside potentially unfavorable ones, while *Lm. fermentum* of vaginal origin may potentially have a role in the gut-vagina axis. In conclusion, our results strongly support the development of multispecies and multistrain probiotic blends, as the combined metabolic cooperation of vaginal lactobacilli may offer greater efficacy for vaginal health compared to single-strain supplements.

**Supplementary Information:**

The online version contains supplementary material available at 10.1007/s00018-026-06107-2.

## Introduction

While the gut and oral microbiome have been extensively studied, the role of vaginal microbiome in women’s health is gaining significant attention only recently. Indeed, recent research efforts highlighted that the vaginal microbiome directly influences the risk of infections, reproductive issues, and other health conditions [1–3]. Human vagina is a complex environment, with a unique microecosystem that changes throughout a woman’s life due to hormonal fluctuations, immune responses, age, and external factors like infections and antibiotics [4, 5]. The healthy adult vaginal microbiota is typically dominated by *Lactobacillus*species, while a more diverse microbial community often indicates compromised health [6]. Large cohort studies allowed to categorize the vaginal microbiome into five Community State Types (CSTs), each characterized by different dominant species: CTS-I is dominated by *Lactobacillus crispatus*, CTS-II by *Lactobacillus gasseri*, CTS-III is associated with transitions and dominated by *Lactobacillus iners* and CTS-V by *Lactobacillus jensenii*. CTS-IV has a more heterogeneous community of strict and facultative anaerobes (*Gardnerella*, *Atopobium*, *Mobiluncus*, *Prevotella*, etc.) and it is often associated with bacterial vaginosis [1, 7–9]. Indeed, an imbalance in the vaginal microbiome, known as bacterial vaginosis (BV), is a common issue, particularly in women during reproductive age, and it is often linked with the dominance of anaerobic bacteria and a reduction in*Lactobacillus *[6]. Other conditions, like vulvovaginal candidiasis (VVC) and streptococcal infections, are also connected with disruptions in the vaginal microbial communities [10]. A dominance of non-*Lactobacillus*species was also found in post-menopausal women, and it was associated with vaginal dryness [11]. Antibiotic treatments for vaginal infections can also have negative side effects, including disrupting the beneficial vaginal microbiota and increasing the risk of recurrent infections and antibiotic resistance. For this reason, scientific and commercial interest towards the development of alternative therapies increased. Vaginal probiotics, administered either topically or orally, are currently promising to restore the balance in the vaginal microbial community and preventing recurrent infections by inhibiting pathogen growth [12]. Since *Lactobacillus* species are crucial for a healthy vaginal environment, there is a growing interest in their role and potential benefits [5]. Indeed, potential probiotic candidates for topical use should show some key attributes, such as promoting vaginal epithelial cell membrane integrity, adhering to the vaginal epithelium, and producing beneficial metabolites [13]. On the other side, while biofilm formation promotes the colonization and persistence of microbial strains in the vaginal environment, if it is not well balanced, it may interfere with sperm motility and fertility [1, 14, 15]. Another important trait is effective glycogen metabolization. Glycogen, abundantly found in vaginal fluid, is metabolized by vaginal lactobacilli, lowering pH and hindering pathogens [16]. On the contrary, an undesired trait is the production of biogenic amines, to prevent malodor and increase in pH, leading to dysbiosis [17, 18].

The use of probiogenomics is revolutionizing the identification of novel probiotic strains [19]. Indeed, a wide genetic profiling of multiple strains may support the discovery of novel potential probiotics, screening in time-consuming and expensive laboratory tests only those strains that have been previously selected in silico. Based on this principle, this study aims to identify new, effective probiotics for improving women’s vaginal health. In particular, we explored the hypothesis that a combined use of multiple lactobacilli species may be more effective. Starting from a genomics screening of vaginal isolates and comparative genomics of these strains with other isolates from the same species, we identified a complementary role of different vaginal species in key activities that may be connected with women’s health, highlighting the promising avenue for the development of a multi-species probiotic product.

## Materials and methods

### Sampling and strains isolation

Vaginal swabs (Clinicswab^LTS^, APTACA S.p.A.) were self-collected by fifteen fertile and ten menopausal women (18–55 years old). The eligibility was defined according to the following inclusion criteria: (1) no symptoms of vaginal or urinary tract infection; (2) no menstrual period; (3) no use of local antibiotics and antifungals in the last six months. Women were instructed about the aim of the study and signed a written consent to participate. Women who did not meet the inclusion criteria were excluded from the study.

Swabs were striked on De Man, Rogosa and Sharpe agar (MRS, Oxoid) supplemented with 0.05% L-cysteine for lactobacilli isolation [20]. Plates were incubated anaerobically for 48 h at 37 °C. Morphologically different colonies were isolated. The isolates that were Gram-positive, catalase negative and rod-shaped were considered. Isolates were preliminary identified using MALDI-TOF Biotyper Sirius (Bruker), according to the manufacturer’s standard protocol [21].

### Isolate deduplication, genome sequencing and analysis

Isolates from the same species were deduplicated using (GTG)_5_-rep-PCR fingerprinting [22]. DNA from the colonies was extracted using the Wizard^®^ Genomic DNA Purification Kit (Promega). After this step, 20 strains were kept for the following analyses (Table 1). Whole Genome Sequencing (WGS) was carried out on Illumina NovaSeq platform, leading to 2 × 150 bp, paired-end reads. Reads were assembled through the SPAdes pipeline (version 4.1.0) [23] and contigs > 1000 bp were filtered using Seqtk [24]. Taxonomic identification was obtained using PhyloPhlan 3.0 [25].

**Table 1:** Taxonomic identification of vaginal strains isolated in this study

Strain origin	Strain	Probiotical Internal Collection Code	MALDI-TOF identification	WGS identification
This study	E3	770/2	*Lactobacillus crispatus*	*Lactobacillus crispatus*
This study	T1	770/4	*Lactobacillus crispatus*	*Lactobacillus crispatus*
This study	U2	ID 2026	*Lactobacillus crispatus*	*Lactobacillus crispatus*
This study	Z1	770/6	*Lactobacillus crispatus*	*Lactobacillus crispatus*
This study	K1	770/3	*Lactobacillus crispatus*	*Lactobacillus crispatus*
This study	X2	770/5	*Lactobacillus crispatus*	*Lactobacillus crispatus*
Probiotical collection	ID_1873	DSM 24619	*Lactobacillus crispatus*	*Lactobacillus crispatus*
Probiotical collection	ID_1626	DSM 24439	*Lactobacillus crispatus*	*Lactobacillus crispatus*
This study	S1	ID 2028	*Lactobacillus gasseri*	*Lactobacillus gasseri*
This study	B1	772/3	*Lactobacillus gasseri*	*Lactobacillus gasseri*
This study	A1	770/7	*Lactobacillus gasseri*	*Lactobacillus gasseri*
Probiotical collection	ID_1969	DSM 32405	*Lactobacillus gasseri*	*Lactobacillus gasseri*
This study	R3	770/9	*Lactobacillus paragasseri*	*Lactobacillus paragasseri*
This study	J1	ID 2029	*Lactobacillus paragasseri*	*Lactobacillus paragasseri*
This study	J2	770/11	*Limosilactobacillus fermentum*	*Limosilactobacillus fermentum*
Probiotical collection	ID_686	DSM 32277	*Limosilactobacillus fermentum*	*Limosilactobacillus fermentum*
Probiotical collection	ID_1460	DSM 18297	*Limosilactobacillus fermentum*	*Limosilactobacillus fermentum*
Probiotical collection	ID_1853	DSM 26956	*Limosilactobacillus fermentum*	*Limosilactobacillus fermentum*
Probiotical collection	ID_1637	DSM 19187	*Limosilactobacillus fermentum*	*Limosilactobacillus fermentum*

### Phylogenetic analysis

We included in the comparative analysis publicly available genomes of the four isolated species, namely *Lactobacillus crispatus* (*n* = 396), *L. gasseri* (*n* = 62), *L. paragasseri* (*n* = 32) and *Limosilactobacillus fermentum* (*n*= 156). Genomes of these species were downloaded from the NCBI database [26]. We manually retrieved the “isolation source” from NCBI or from the original study (Online Resource1).

Phylogenetic tree for each species (including new isolates and NCBI genomes) was developed using the PhyloPhlAn 3.0 pipeline [25] and visualized using iTOL v6 [27]. The Average Nucleotide Identity (ANI) values between each pair of genomes was calculated using fastANI (version 1.0) [28]. Classical Multidimensional Scaling (MDS, *cmdscale* R function) was carried out on the ANI distance matrix and plots were visualized using the *ggplot2* R package.

### Pangenome and gene onthology (GO) analysis

Coding sequences (CDS) of each genome were predicted using Prokka (version v1.11) [29]. Pangenomes of each species was computed using the Roary pipeline [30]. Then, we used the Gene Ontology database [31], to search for the key GOs in core/soft-core and shell/cloud genes of the different species using UNIPROT 2023_04 [32], including in the analysis only genomes from vaginal source. GOs terms of interest for vaginal lactobacilli were considered as described in the supplementary material of the work by Bhattacharya et al. [13].

### Functional analysis through the alignment of predicted genes against protein reference sequences

Predicted genes in the analyzed genomes were aligned against key enzymes of vaginal interest using DIAMOND blastx (version 2.1.11) [33]. A hit was considered when displaying at least 80% of identity on at least 50% of the query length. Protein sequences of key genes were downloaded from NBCI. Genes searched included those related to the adaptation in the vaginal niche, to the production of beneficial metabolites and to the resistance to gastrointestinal passage, selected through screening of the scientific literature using different combinations of keywords in SCOPUS search: ‘vaginal adaptation microbial enzymes’, “*Lactobacillus* role in women’s health”, “vaginal fitness”, “glycogen metabolism”, “vaginal health”, “pathogen inhibition”, “genomic analysis”, “vaginal dysbiosis”, “sialidase activity”, “biofilm formation”, “immune modulation”, “probiotic for vaginal health”, “probiotic genetic traits”, “*Lactobacillus crispatus* enzymes”, “adhesion disruption enzymes vaginal bacteria”, “microbial degradation of vaginal mucus”, “adhesion to vaginal epithelium”, “bacterial vaginosis”, “bacteriocin production”, “folate metabolism”, “tryptophan metabolism”. Genes included in the custom database and NCBI accessions are reported in Online Resource 2.

The ability of genomes to degrade different carbohydrates sources was estimated by aligning predicted genes against the Carbohydrate-Active Enzyme database [34]. The presence of known antibiotic resistance (AR) genes involved in resistance to antibiotics was evaluated using TORMES, that search against CARD, AMRfinder and ARGANNOT databases considered [35]. The presence of plasmids in the newly sequenced genomes was verified using Plasmid Finder Tool [36].

Heatmaps were obtained from presence-absence gene matrices using the R package ‘pheatmap’.

## Results

### Comparative genomics reveals genomic adaptation to specific environmental niches

Isolate identification obtained by MALDI-TOF [21] was confirmed using WGS, that is the final one considered for further analyses (Table1).

**Fig. 1:**
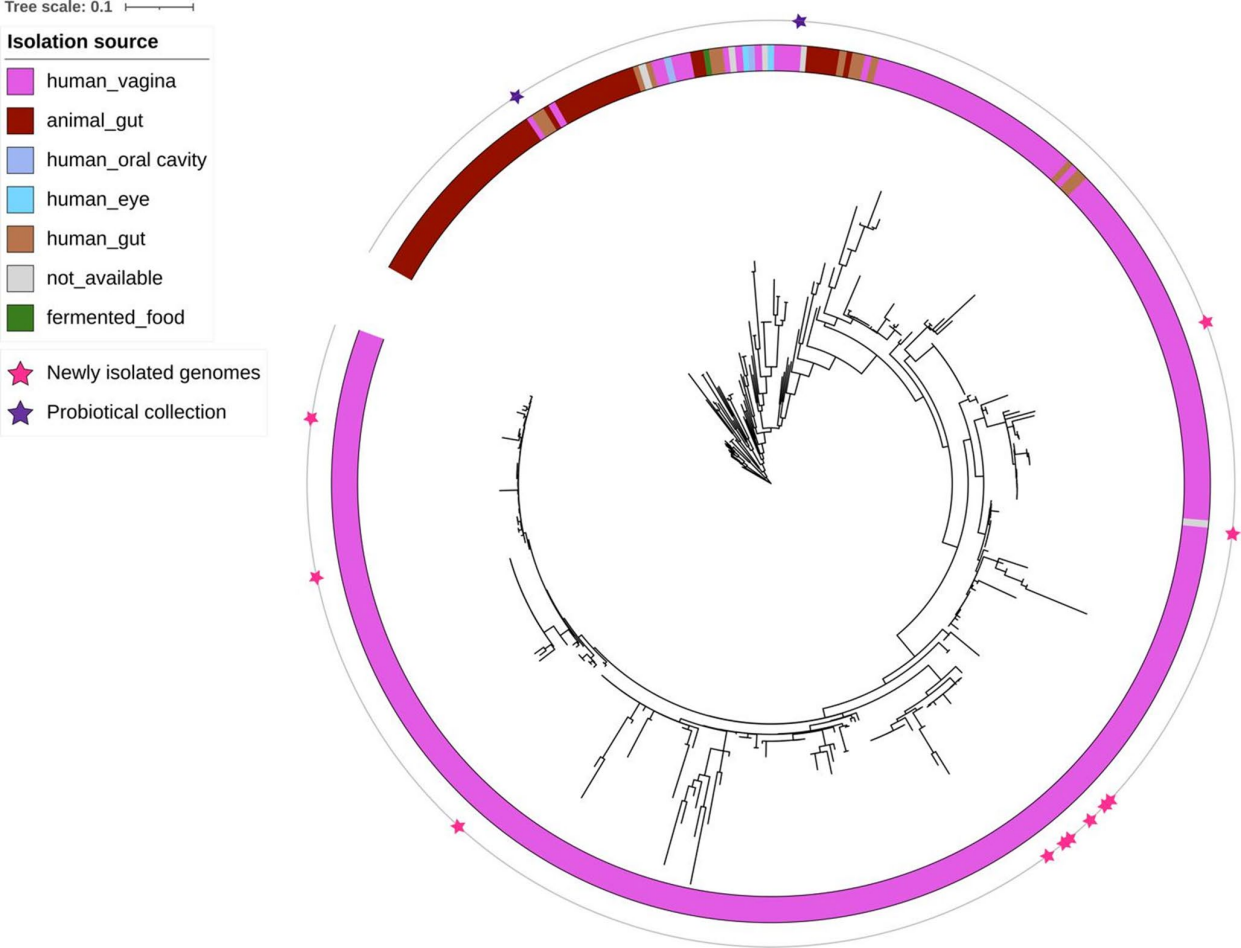
Phylogenetic tree of *Lactobacillus crispatus* genomes. The outer ring is coloured according to the isolation. The genomes newly isolated in this study and those provided by Probiotical are indicated by stars

Through a phylogenetic analysis, although most of *L. crispatus* genomes available are of vaginal origin, we can see that the host (human or animal) drives the clustering of the strains (Fig. 1). In addition, we observed 3 different genome clusters within *L. crispatus*species, suggesting the presence of putative different sub-species, as previously reported [37]. Two clusters exclusively included strains of vaginal source, while the other comprised strains from mixed isolation sources. Interestingly, strains newly isolated in this study fell in both the sub-species.

We found that several *L. gasseri/L. paragasseri*genomes present in NCBI were mislabeled and positioned inconsistently into the phylogenetic tree. Therefore, for the final identification of NCBI strains of these species, we referred to that reported by Ene et al. [38].

**Fig. 2:**
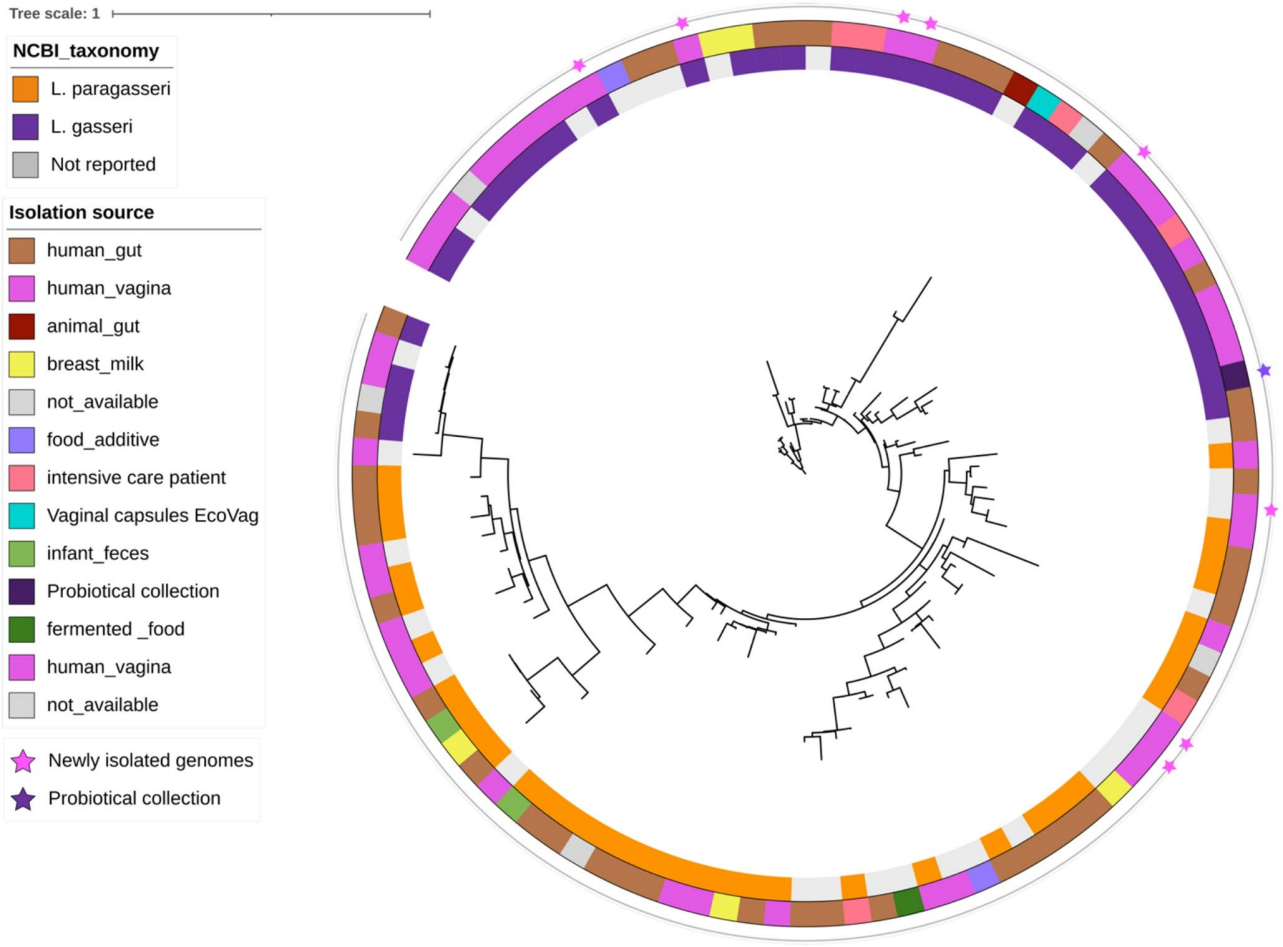
Phylogenetic tree of *Lactobacillus gasseri* and *Lactobacillus paragasseri*genomes. The inner ring is coloured according to the species-level identification (as reported by Ene et al. [38]). The outer ring is coloured according to the isolation source The genomes newly isolated in this study and those provided by Probiotical are indicated by stars

Indeed, in Fig. 2 we can clearly see the separation of the genomes of *L. gasseri* and *L. paragasseri* on the tree. However, we did not find a clear genome clustering according to the isolation source within these species (Fig. 2).

**Fig. 3:**
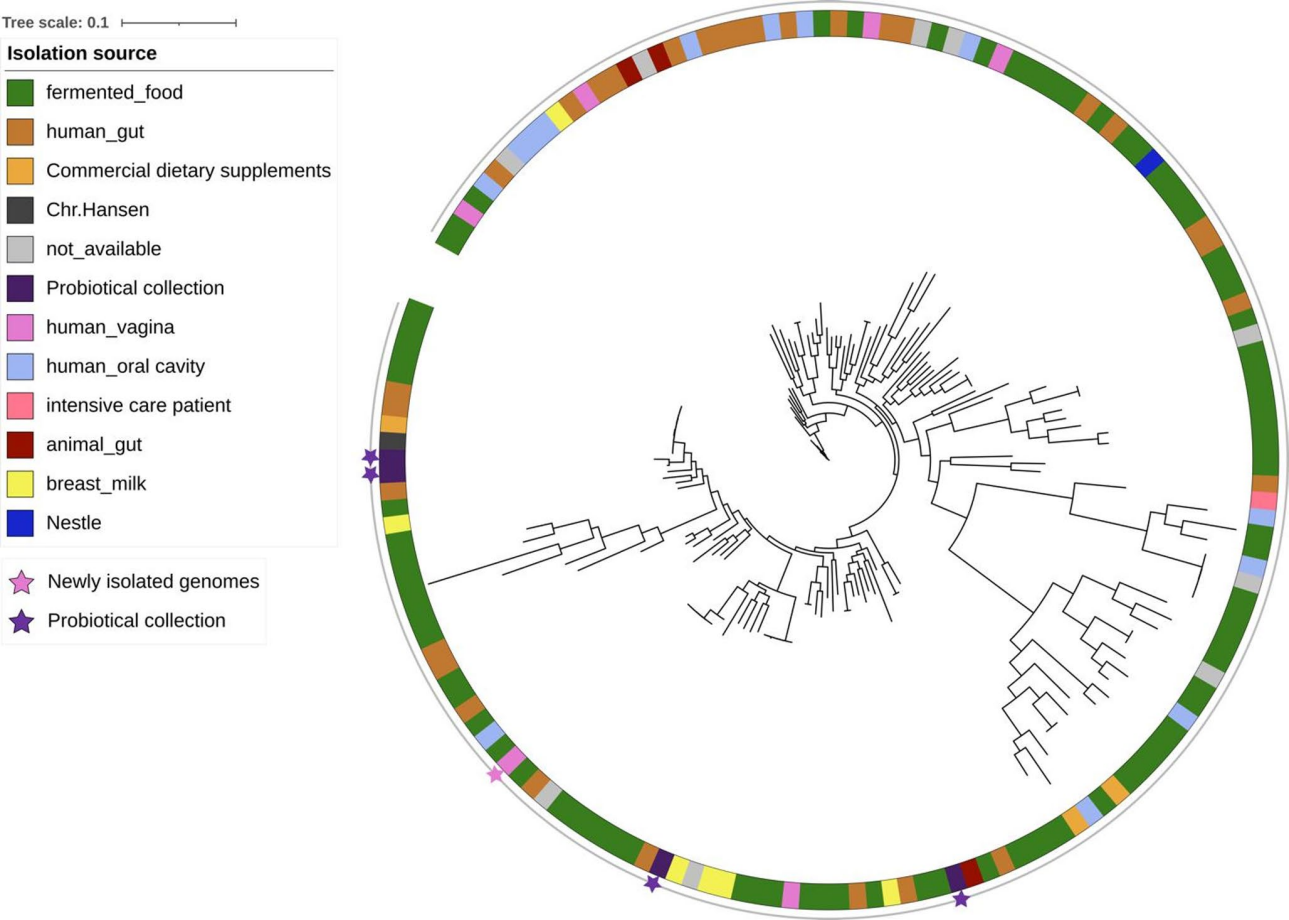
Phylogenetic tree of *Limosilactobacillus fermentum.* The ring is coloured according to the isolation source The genomes newly isolated in this study and those provided by Probiotical are indicated by stars

Finally. we did not observe a genome clustering driven by the strain origin for Limosilactobacillus fermentum, (Fig. 3). However, it has to be pointed out that the dataset included only few genomes of vaginal source, (*n* = 5 out of 156; 1 newly isolated in this study)

### Pangenomic insights demonstrate different genetic evolutionary dynamics in the main vaginal lactobacilli species

The pangenome refers to the complete set of genes present in all individual strains or isolates of a particular species. This gene pool is divided into two main categories: core genes, which are shared by all the members of the species, and accessory genes, which are present in only some individuals.

**Fig. 4:**
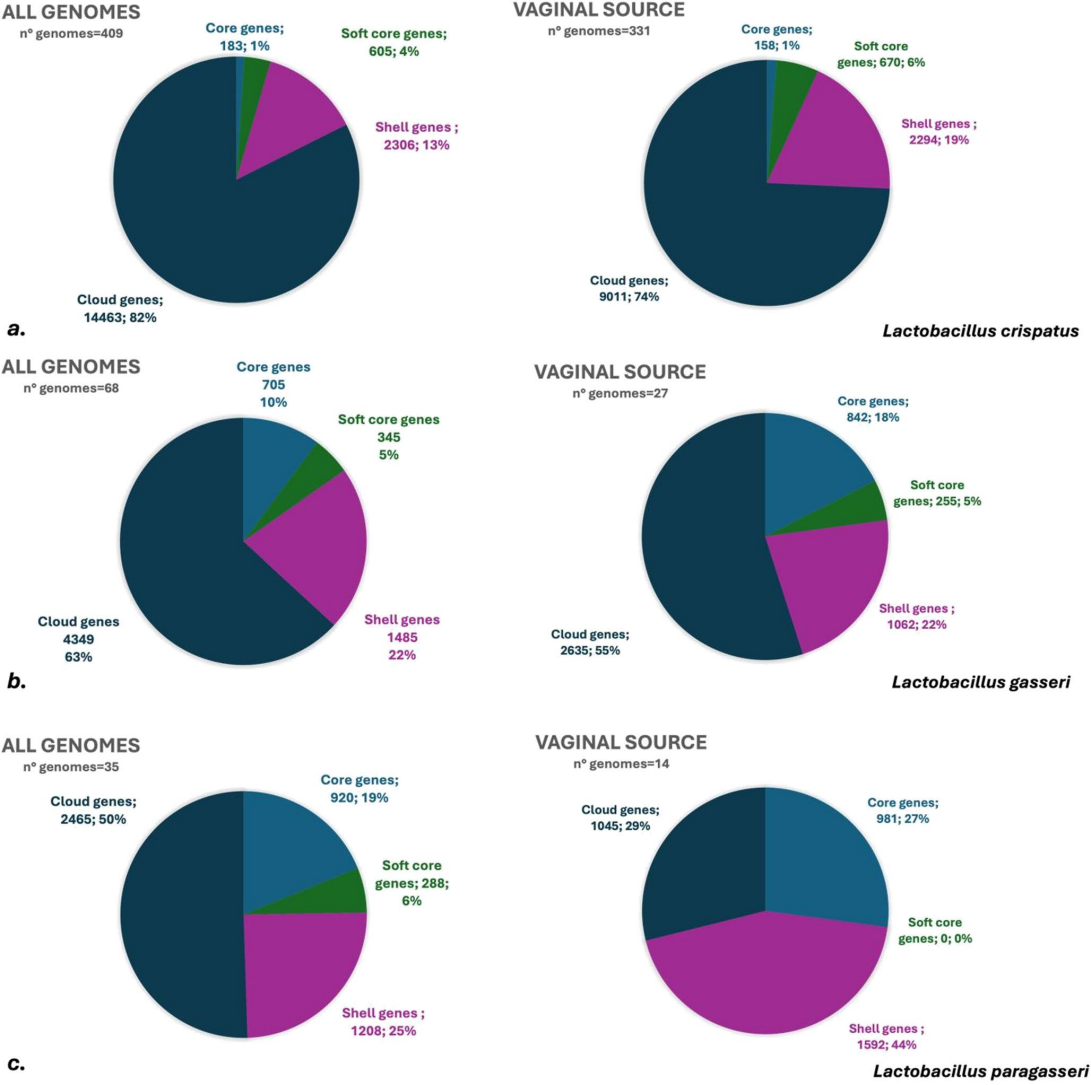
Comparison and distribution of the percentage of core, soft-core, shell and cloud genes in the species *L. crispatus* (**a**) *L. gasseri* (**b**) and *L. paragasseri* (**c**) from vaginal origin

A single genome sequence captures a specific moment in the history of a lineage. While some genes and mutations have a long-shared history and are predisposed to persist within a single species over generations, others are temporary, and the gene loss/acquisition events may be boosted by environmental factors [39]. In the pangenome analysis, genomes from *L. crispatus* (*n* = 396; 13 newly isolated in this study), *L. gasseri* (*n* = 62; 6 newly isolated in this study), *L. paragasseri* (*n* = 32; 3 newly isolated in this study) were included. In order to exclude biases due to the different isolation sources of the strains considered, we further calculated the pangenome considering exclusively genomes of vaginal origin (Fig. 4). For this reason, we excluded the genomes of *Lm. fermentum*, since only 5 genomes of vaginal origin were present in NCBI.

We observed that *L. paragasseri* retained the highest percentage (25%) of core/soft-core genes (defined as present in = > 99–100% and 95%−99% of the genomes, respectively), while *L. crispatus* retained the lowest number of core genes (core + soft-core; 5%) (Fig. 4). Consistently, *L. crispatus* had the highest percentage (82%) of cloud genes, that is genes present in < 15% of the genomes considered, although this may be affected by the higher number of genomes available for this species. Considering only the genomes of vaginal origin, an increase in core genes was observed for *L. gasseri* and *L. paragasseri*, suggesting the presence of niche-specific functions, related to the adaptation to the vaginal environment. As expected, the situation remained unchanged for *L. crispatus*, since 81% of the genomes included in this study were of vaginal origin (Fig. 4).

The distributions of key GOs within the pangenomes are summarized in Online Resource 3, while a full list of the key GOs found in the pangenomes is reported in Online Resource 4. From the intersection of the core genes of the three species (Online Resource 3), we did not find any GOs relevant for the vaginal environment (among those highlighted by Bhattacharya et al. [13], but rather the three species only share primary biological functions (e.g., ATP hydrolysis activity, proteolysis, glycolytic process, etc.). In the core of *L. paragasseri* strains, we found the biosynthesis of fatty acids, associated with the integrity of the vaginal cell membranes [40]. In *L. gasseri* core, the biosynthesis of organic acids and hydrogen peroxide were identified, that are relevant for the antimicrobial and anti-pathogenic action. Finally, the core genome of *L. crispatus* is characterized by the GO related with bacteriocin biosynthesis.

Differently, in the cloud of *L. gasseri* and *L. paragasseri* (Online Resource 3) we found key GOs that are related with adhesion, aggregation and synthesis of exopolysaccharides, while the three species share in their cloud genome hydrolase and transferase activities involved in the synthesis of biosurfactants, that may help preventing microbial infections.

### Functional screening highlights genomic traits linked with fitness in the vaginal niche and species-specific mechanisms

**Fig. 5:**
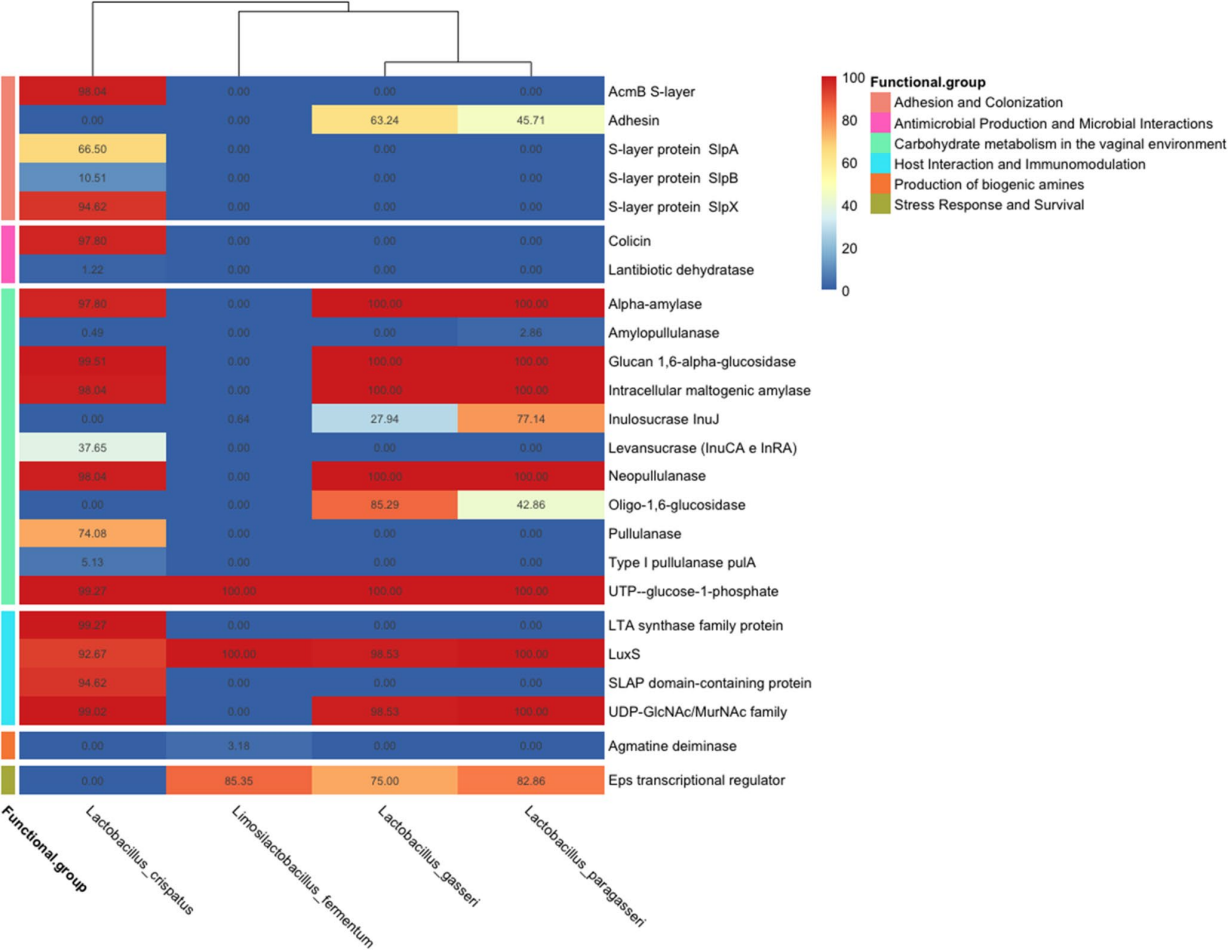
Prevalence (%) of genes associated with vaginal adaptation in the genomes of lactobacilli

We firstly screened genomes of vaginal origin for the presence of genes related to the adaptation and persistence in the vaginal environment, grouped in the following functional classes: (i) Adhesion and Colonization; (ii) Antimicrobial Production and Microbial Interaction; (iii) Carbohydrate metabolism in the vaginal environment; (iv) Host interaction and Immunomodulation; (v) Stress Response and Survival (Fig. 5). Twenty-four (out of 59) of the genes included in the manually curated database (Online Resource 2) were found. *L. crispatus* genomes have the highest prevalence of these genes (12 out of 24 genes were present in > 93% of the genome), as well as the largest number of genes involved in immunomodulation, adhesion to cervicovaginal epithelial cells and antagonism against pathogens of the urogenital tract. This support the observation that *L. crispatus* dominated CS are the most frequent in women and are associated with a general vaginal health. We observed a very similar profile in *L. gasseri* and *L. paragasseri* in the context of vaginal adaptation. As shown in Fig. 5, the two species have a high genomic overlap, confirming a close evolutionary relationship [41]. It is therefore interesting to investigate the differences at the strain level. Both species have a lower gene repertoire than*L. crispatus* for activities potentially linked with host interaction and immunomodulation. Similarly, these two species have fewer genes, among those examined, involved in the adhesion and colonization processes. In contrast, the gene encoding the transcriptional regulator Eps has been identified in the genomes of *L. gasseri* and *L. paragasseri*, while it is absent in *L. crispatus*. On the contrary, the lowest prevalence of genes was observed in *Lm. fermentum* genomes, where all the genes found were those involved in biofilm formation and co-aggregation. In addition, genes related to the production of biogenic amines, such as cadaverine, putrescine and trimethylamine, that are a negative trait for a vaginal strain, since are responsible for the increase of vaginal pH, promoting dysbiosis and unpleasant odors, were not found in the genomes screened.

Among the enzymes involved in bacterial carbohydrate metabolism in the human vagina, the absence of sialidase stands out in all the species analyzed. Bacterial sialidase degrades glycans in vaginal mucus by removing sialic acid. The presence of this enzyme may offer limited nutritional benefits for vaginal lactobacilli but carries the risk of compromising the integrity of the protective mucus; its absence promotes mucus stability but limits access to specific nutritional sources.

**Fig. 6:**
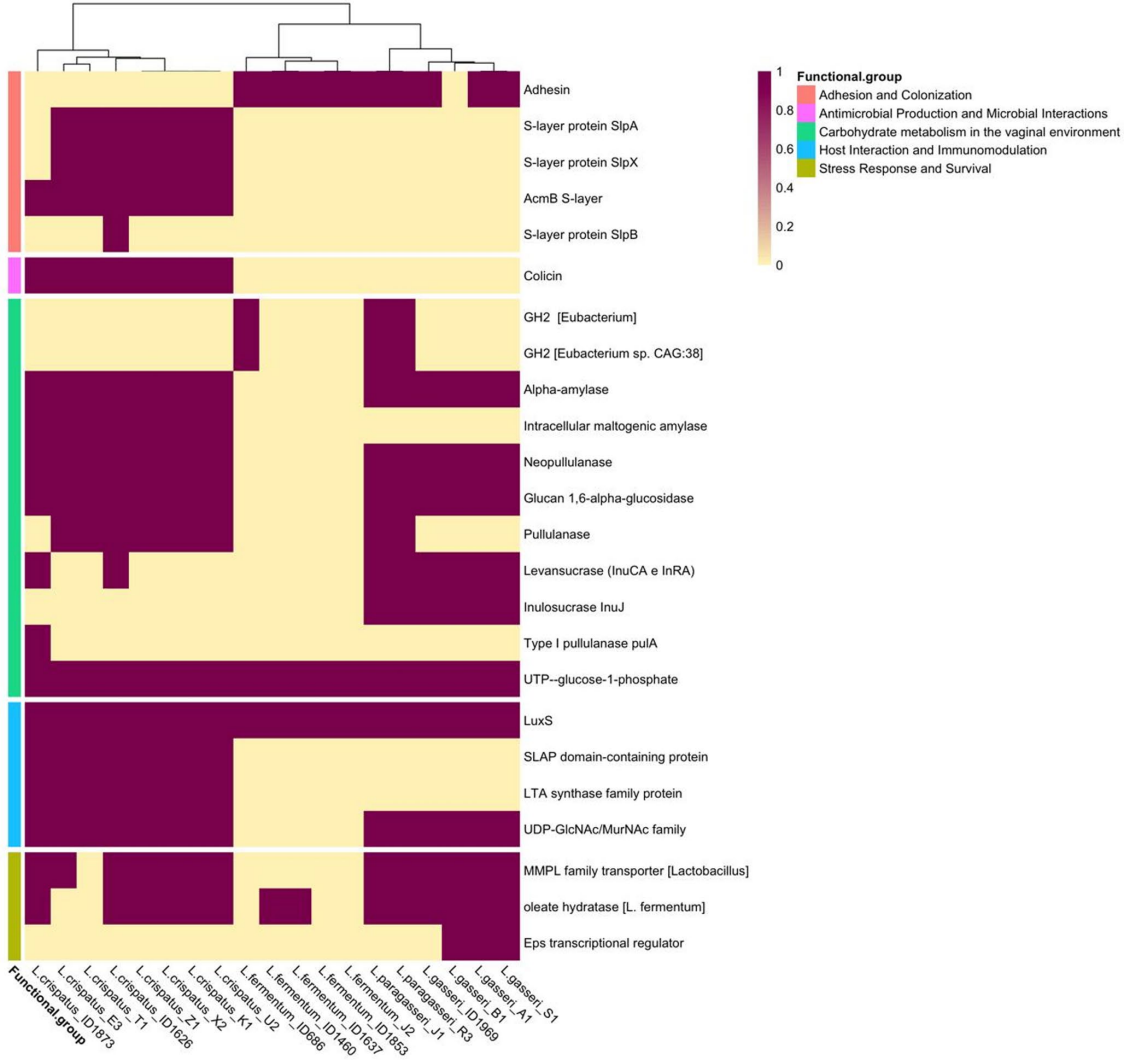
Prevalence of genes associated with vaginal adaptation in the genomes of the newly isolated strains

We screened the newly isolated genomes for the presence of the 59 genes involved in vaginal adaption (Online Resource 2) (Fig. 6). We confirmed that our *L. crispatus* strains exhibited the highest prevalence of the genes searched. *L. gasseri* and *L. paragasseri* displayed a highly similar gene profile. Consistently with previous observations, *Lm. fermentum* presented the lowest prevalence of the analyzed genes, particularly those associated with biofilm formation and co-aggregation. Variability was observed in the presence of genes involved in Carbohydrate Metabolism in the Vaginal Environment and Stress Response and Survival (Fig. 6). This suggests distinct mechanisms of adaptation to the potentially dynamic conditions of the vaginal milieu and indicates that each species may apply slightly different metabolic strategies for utilizing available resources. The absence of sialidase-related genes across all strains was noted, suggesting that direct degradation of vaginal mucus via this enzyme may not be a common strategy among these lactobacilli. In addition, genes related with the fatty acid response mechanism, important when vaginal bacterial vaginosis may be treated with oleic acid as alternative to antibiotics [42], were found in all the strains.

### *Lactobacillus crispatus* and *Lactobacillus gasseri* are good candidates as oral probiotics targeting women’s health

**Fig. 7:**
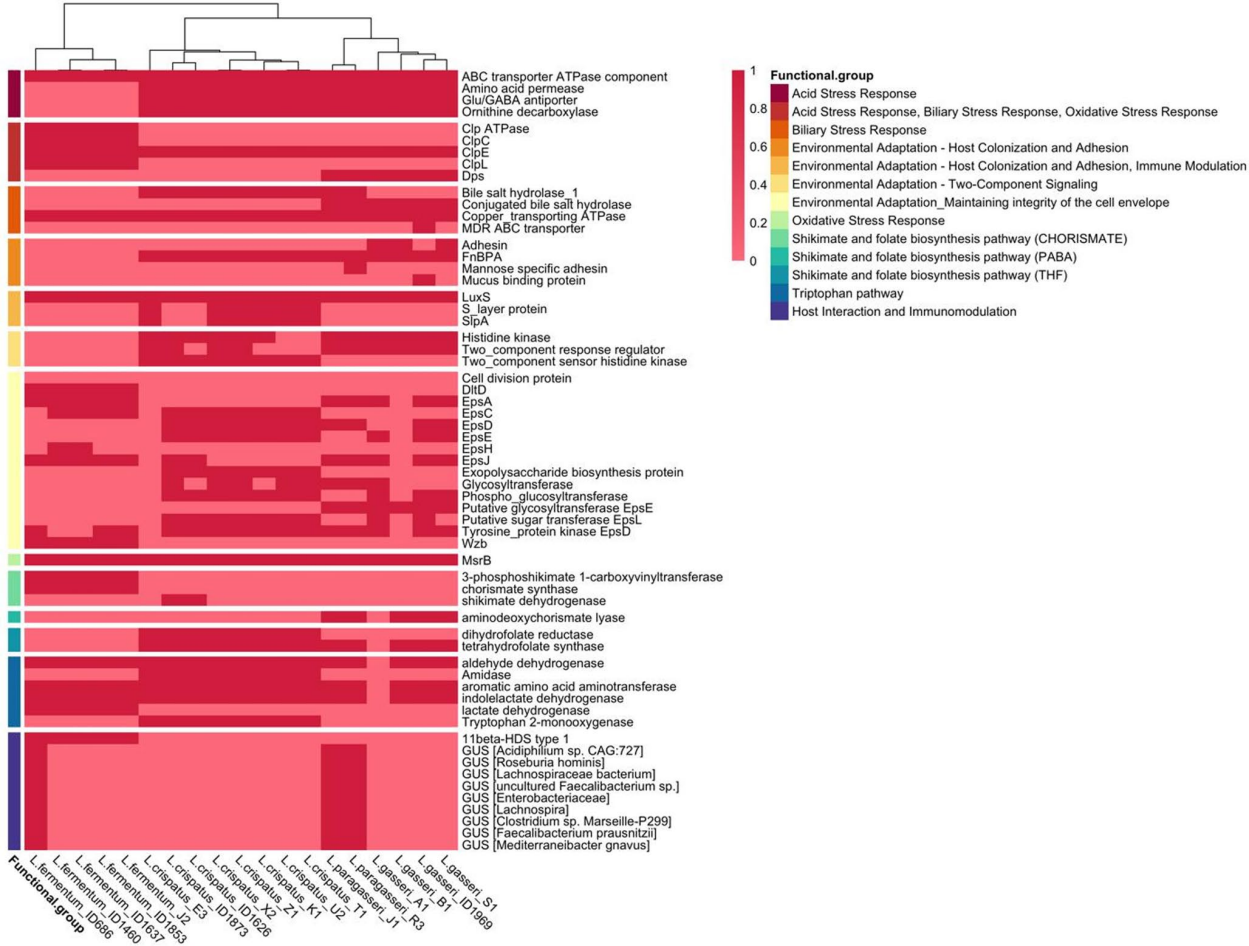
Occurrence of genes associated with probiotic factors in the genomes of the strains newly isolated in this work

We further screened the genomes of newly isolated strains for the presence of genes related to key probiotic factors, assuming a potential oral use of our strains. The genes screened included those for the resistance to stress and gastrointestinal (GIT) passage and the production of beneficial metabolites in the gut (e.g., folate and tryptophan metabolism) (Fig. 7). To reach the colon, oral probiotics must be able to survive gastric acidity, bile salts in the small intestine, and oxidative stress, through a variety of resistance mechanisms or active stress responses. Many of the analyzed strains harbour genes involved in resistance to acid stress (broadly present in all except *Lm. fermentum* genomes), biliary stress (present in *L. crispatus* and *L. paragasseri*, but absent in *L. gasseri* and *Lm. fermentum*), and oxidative stress (methionine sulfoxide reductase *MsrB* reverses the loss of the biological activity of proteins caused by the oxidation of methionine to methionine sulfoxide and, therefore, contributes to the protection of bacteria against oxidative damages, and it was present in all the genomes). Although all the strains potentially have genes involved in different resistance mechanisms, *Lm. fermentum* strains were those showing the lowest occurrence, thus potentially performing worse during GIT passage.

We also explored the potential of the strains to produce folates, particular important for women during pregnancy. Chorismate is an important biochemical intermediate, as it acts as a precursor in the biosynthesis of aromatic amino acids, but also of indole, indole derivatives, tryptophan and folic acid [43]. The genes coding for 3-phosphoshikimate-1-carboxyvinyltransferase and chorismite synthase, final enzymes of the shikimate pathway that leads to chorismate production, were observed exclusively in *Lm. fermentum* genomes (Fig. 7). On the contrary, all the strains, except *Lm. fermentum*, had the gene coding for tetrahydrofolate synthetase, involved in the production of tedrahydrofolate (THF), while dihydrofolate reductase and shikimate dehydrogenase were exclusively found in *L. crispatus*. The first one is involved in the final step of tetrahydrofolate biosynthesis, which is the active form of folic acid for humans, while the second one catalyzes the fourth step of the shikimate pathway that leads to the production of precursors necessary for folate biosynthesis in bacteria and is absent in mammals. *L. gasseri* and *L. paragasseri*strains share the aminodeoxychorismate lyase gene, which leads to the production of para-aminobenzoic acid (PABA), an important intermediate in the bacterial synthesis of folate [43].

Another important feature is the synthesis of the indole metabolites of tryptophan (Fig. 7), which are important both at intestinal (involved in immune modulation, barrier integrity, bacterial communication, protection from stress) and at systemic (benefits on mental health, since they are involved in serotonin and melatonin biosynthesis) [44]. *L. gasseri* A1, *L. crispatus* C3 and *L. crispatus* Q1 did not show any of the mapped genes involved in tryptophan metabolism. All other genes were present in all the genomes except the lactate dehydrogenase, which was present only in *Lm. fermentum* genomes, indicating that only this strain harbored the complete pathway of indolic 3-lactic acid (ILA) production (Fig. 7). Tryptophan 2-monooxygenase, leading to the production of indole-3-acetamide (IAM), was present only in *L. crispatus* genomes and, coupled with the presence of amidase and aldehyde dehydrogenase, complete the metabolic pathway for indolic 3-acetic acid (IAA) production in these strains.

We further screened the genomes for the presence of genes related with beneficial or detrimental activities for women health, possibly explicated in the human gut, thus supporting their oral administration. GUS (β-glucuronidase) genes code for an enzyme that deconjugate estrogens and are involved in female estrobolome and hormonal disorders [45], were found in all the newly isolated *L. paragasseri* strains and in *Lm. fermentum*ID 686, while were absent in other strains. Moreover, hydroxysteroid dehydrogenase (HSD) genes [46], involved in steroid degradation, were observed only in *Lm. fermentum* strains.

No known gene potentially involved in antibiotic resistance were found in lactobacilli strains isolated in this study. In addition, we screened the genomes for the presence of plasmids. Plasmids were detected in 12 out of 19 genomes (Online Resource 5). However, mapping genes present on plasmids against our database of key genes involved in the vaginal fitness, we did not find any matches, suggesting that these activities are well conserved among vaginal-origin lactobacilli.

### Lactobacilli are equipped with enzyme families that are important for the adaptation and survival in the vaginal niche

**Fig. 8:**
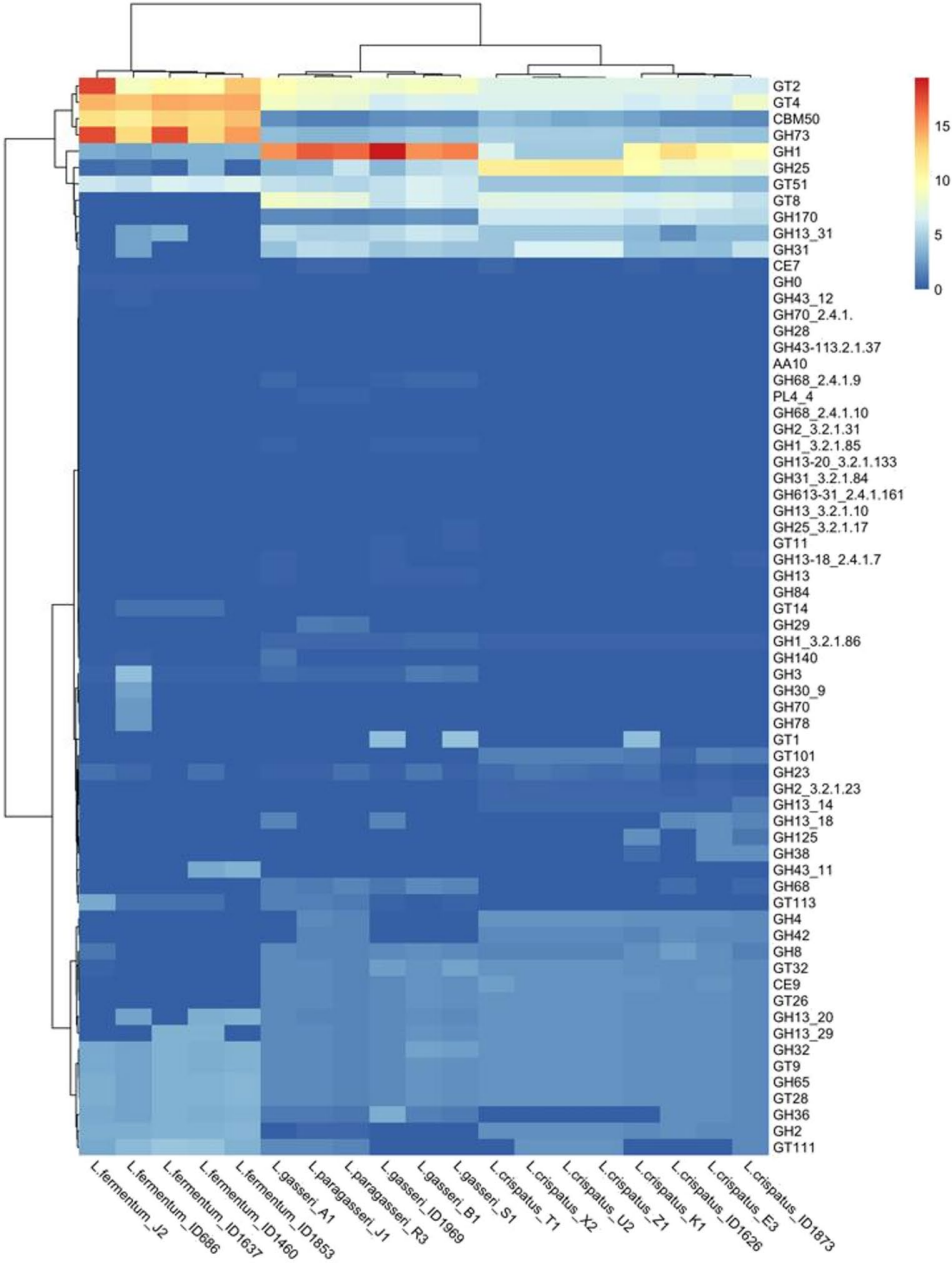
CAZymes prevalence in the genomes analysed in this study The color scale indicates the % of genes from each CAZy family found in each genome

We screened the strains for the ability to degrade carbohydrates, searching for Carbohydrates-Active genes (CAZy). Sixty-six CAZy families were identified, 11 of which showed a higher number of genes present: CBM50, GH1, GH13_31, GH25, GH31, GH73, GH170, GT2, GT4, GT8, GT51 (Fig. 8). In the analyzed genomes, the identified CAZy families are predominantly associated with the processing of complex carbohydrates, including structural and storage polysaccharides such as cellulose, chitin, starch, glycogen and peptidoglycan, as well as glycoconjugates. In the context of the vaginal microbiome, GH1 and GH31 families are important for the degradation of glycogen, an energy source for lactobacilli. GH73 and GH25 families act on bacterial peptidoglycan, potentially influencing interactions among different strains sharing the same environmental niche and GH170 includes enzymes which act on particular bonds present in mucins, thus may degrade components of the vaginal mucus. Finally, GT2 and GT4 (including glycosyltransferases that catalyze the polymerization of bacterial exopolysaccharides) are involved in the synthesis of polysaccharides that may influence bacterial adhesion and biofilm formation in the vagina. These enzyme families are therefore relevant for the adaptation and survival of microorganisms in the vaginal niche.

We observed a species-specific pattern in the distribution of CAZymes: for example, genes that fall into the GT2, GT4, and GH73 families were prevalent in the genomes of *Lm. fermentum*. On the contrary, the CAZy GH1 family was particularly abundant in *L. gasseri* and *L. paragasseri*, while in the genomes of *L. crispatus* we found genes that fall into the GH25 family (Fig. 8).

## Discussion

The vaginal microbiome has a direct impact on infections, reproductive health and other women’s health issues. A vaginal microbiota dominated by *Lactobacillus* is generally indicative of a healthy environment, while higher microbial diversity often indicates dysbiosis, which has led to a growing interest in the role and benefits of *Lactobacillus*in this context. Modulation of vaginal microbiome through the administration of probiotics shows promise in restoring balance and preventing recurrent infections. Genomic screening of putative probiotic strains supports a faster development of well-targeted and personalized probiotic formulations. However, some important features related to probiotic activities may be highly niche-specific, thus suggesting that strains of vaginal origin may perform better in that specific environment than probiotics of different origin. Indeed, although several probiotics targeted to women’s health are available on EU market [47], several of them contain strains from different isolation sources.

In this study, we isolated strains from four different lactobacilli species, namely *L. crispatus*, *L. gasseri*, *L. paragasseri*, and *Lm. fermentum*. Notably, two other vaginal species, *L. iners* and *L. jensenii*, were not found. This is likely due to the limited sample size and the lower prevalence of these species in the vaginal environment, together with a possible bias during isolation, due to the choice of the culture medium. Specifically, *L. crispatus* and *L. gasseri*/*L. paragasseri* are known to dominate the vaginal Community State Types (CST-I and CST-II, respectively), often representing > 50% of the microbial population. *L. jensenii*, while typical of a healthy vaginal microbiota, is present in approximately 20% of the women, suggesting that a larger cohort would have been necessary for its isolation [48, 49]. We also failed in isolating *L. iners*, usually associated with less stable or transitional vaginal communities and frequently linked with vaginal dysbiosis [47–49]. While*Lm. fermentum*is not typically a dominant species in the gastrointestinal tract or in the vagina, its established probiotic characteristics make it a promising candidate [48, 50–52]. In our study we demonstrated that for *L. crispatus*, and to a lower extent for *L. gasseri* and *L. paragasseri*, a clear genomic adaptation to the vaginal environment occurred. This was not the case for *Lm. fermentum* strains, although this result may be influenced by the limited number of genomes from strains of vaginal origin available. This was further supported by the pangenome analysis, that showed that *L. crispatus*retained the lowest number of core (core + soft-core) and the highest number of cloud genes, which suggest the evolutionary diversification of these strains, possibly associated with the adaptation to different niches. Its genome plasticity and ability to evolve may explain why this species is the most frequently found in the vaginal environment as dominant [13]. In addition,*L. crispatus* is the one with the highest number of genes involved in the adhesion to cervicovaginal epithelial cells and antagonism towards urogenital tract pathogens, further highlighting the high genomic specialization of this species to the vaginal niche and supporting the possible topical use of these strains. Consistently with a previous work on *L. gasseri* and *L. crispatus*strains isolated from human urogenital and gastrointestinal tracts [53], our findings suggest that future probiotic formulations addressed to women’s health should consider the isolation source and carefully evaluate the genetic and phenotypic characteristics of the strains for targeted application in different body sites.

However, a bidirectional communication between the female reproductive system (FRT) and various other organs (e.g., vagina-gut, vaginal-oral, vaginal-brain axes) has been highlighted [54]. Evidence supports the translocation of gastrointestinal *Lactobacillus *strains to the vagina (gut-vagina axis), suggesting that oral probiotic intake may be directly linked with improved vaginal microbiota balance and local immune responses [55]. Direct bacterial translocation from the gut to the vagina has been previously observed [56]. Beyond direct translocation, indirect interactions involving metabolites or immunoglobulin production, as well as the ability of gut microbes of metabolizing estrogens may be also implicated in the gut-vagina communication [52, 57–59].

In order to evaluate the possibility of using our strains in a probiotic blend with oral delivery, we assessed the presence of genomic traits related to GI resistance. All the *L. crispatus*,* L. gasseri* and *L. paragasseri* strains have several genes related with active stress response mechanisms required to survive during gastrointestinal transit, except *Lm. fermentum*strains, which lack some of them. We further screened the genomes for the presence of genes related with beneficial or detrimental activities for women health and possibly explicated in the human gut, thus supporting their role as oral probiotics. For this aim, we searched for the presence of β-glucuronidase, that may deconjugate estrogen metabolites in the gut. This reactivation allows unbound estrogen to be recirculated through the bloodstream, leading to increased reabsorption of free estrogen and increased risk of cancers and hormonal disorders [45]. These genes were only present in all *L. paragasseri* and in one strain of *Lm. fermentum*. Although further in vitro and in vivo studies are necessary to explore the role of β-glucuronidase in women estrobolome, these strains may be potentially involved in hormonal disorders [46].

The production of beneficial tryptophan metabolites and folate are important traits for probiotics targeting women’s health. Given the prevalence of folate deficiency, particularly during pregnancy, there is growing interest in developing probiotics capable of synthesizing folate directly in the gut [43]. Our results indicate that none of the newly isolated strains contain the complete pathway for folate biosynthesis. However, each strain possesses one or more key enzymes for producing important intermediates like PABA, chorismate, and THF. This suggests a complementary metabolism among the strains, which supports their potential use in a multi-species probiotic blend rather than as individual strains. Concerning the synthesis of indole metabolites of tryptophan and the two known pathways, only*Lm. fermentum* genomes show the complete ILA pathway, while *L. crispatus*may synthesize these metabolites through the IAA pathway. The indole derivatives are known to support gastrointestinal homeostasis by maintaining the integrity of the intestinal barrier and modulating anti-inflammatory signaling [60]. Although widely studied in the intestine, their role in the vagina is an emerging field. Evidence suggests that vaginal indoles, including ILA and indole-3-aldehyde, activate the aryl hydrocarbon receptor (AhR) to modulate immune responses and provide protection against vulvovaginal candidiasis [61, 62]. These findings collectively provide a strong rationale for further exploring the role of bacterial tryptophan metabolites, including ILA and potentially IPA, in maintaining vaginal immune homeostasis and preventing dysbiosis. However, we have to point out that this is a preliminary study and several limitations exist, including the relatively small sample size and the possible bias in the strain isolation, due to the choice of the culture medium. Moreover, functional characteristics of the strains are inferred based on genomic data analysis. These predicted functions will need to be validated through in vitro or in vivo experiments in subsequent works. Future studies should also focus on an in-depth analysis of the specific synergistic mechanisms between the different strains (e.g., metabolic complementarity, interactions in gene expression regulation). Finally, the “dual nature” of *L. paragasseri* would deserve further insights to identify which genes may contribute to the adverse phenotypes.

## Conclusion

Recognizing the intrinsic diversity and hormonal variability of the female microbiome, traditional probiotics formulated with generic strains are often suboptimal. For example, hormonal shifts during childbearing years and the decline of estrogen in menopause can significantly alter the vaginal microbiome, increasing the risk of conditions like bacterial vaginosis and urinary tract infections. This highlights the need for targeted probiotic interventions using specific lactobacilli strains capable of effectively colonizing the vagina and providing beneficial activities. Our results demonstrated that strains of vaginal origin are clearly genomically adapted to this niche, giving them higher fitness to persist and counteract pathogens. On the other side, when topical application is not feasible, oral supplementation may also be effective, when selecting the suitable strains. Indeed, we showed that vaginal-origin lactobacilli may potentially survive GI passage and that they may still have beneficial activities in the gut, highlighting the importance of the gut-vagina axis.

Vaginal probiotics containing *L. crispatus* currently represent the most prevalent formulation commercially available. Our findings confirmed the effective performance of this species within the vaginal niche. However, to meet more specific needs for vaginal health, the genetic characteristics of *L. gasseri* can be considered equivalent and complementary. Furthermore, the role of *L. paragasseri* in the vaginal environment presents an intriguing dichotomy: while it exhibits genetic similarities with *L. gasseri*, it is also characterized by potentially unfavorable traits. In summary, *L. crispatus* maintains its primary position due to its robust adaptability to this environment, but the previously underappreciated role of *L. gasseri* emerges, suggesting a potential synergistic interaction between these two species.

Indeed, our results suggest that the combination of multiple species and strains in a probiotic blend may be more effective than a single-strain supplement, since several beneficial activities require metabolic cooperation among the strains.

## Supplementary Information

Below is the link to the electronic supplementary material.

Supplementary Material 1 (PDF 227 KB)

Supplementary Material 2 (PDF 249 KB)

Supplementary Material 3 (PDF 628 KB)

Supplementary Material 4 (PDF 159 KB)

Supplementary Material 5 (PDF 99.5 KB)

## Data Availability

The genomes sequenced in this study are openly available in NCBI, accession numbers PRJNA1297996.
