# Supplementary material for: Women’s health is a team effort: probiogenomics supports the development of a multi-species vaginal probiotic
Source: Cell Mol Life Sci. 2026 Feb 26;83(1):132. doi: 10.1007/s00018-026-06107-2 (PMC12957687; doi:10.1007/s00018-026-06107-2)
Supplement: Supplementary file 1 — Supplementary Material 1 (PDF 227 KB) [file 18_2026_6107_MOESM1_ESM.pdf]

Women's health is a team effort: probiogenomics supports the development of a multi-species vaginal probiotic

Chiara Maria Calvanese[1], Vincenzo Valentino[1], Annachiara De Prisco[2], Serena Allesina[2], Angela Amoruso[2], Francesca Deidda[2], Annalisa Visciglia[2], Danilo Ercolini[1,3], Marco Pane[2], Francesca De Filippis[1,3*]

[1] Department of Agricultural Sciences, University of Naples Federico II, P.zza Carlo di Borbone 1, 80055 Portici (NA), Italy

[2]Probiotical Research S.r.l., via Enrico Mattei 3, 28100 Novara, Italy

[3] Task Force on Microbiome Studies, University of Naples Federico II, Corso Umberto I 43, 80100 Napoli, Italy

Journal: Cellular and Molecular Life Sciences

Corresponding Author:

Prof. Francesca De Filippis

Department of Agricultural Sciences, University of Naples Federico II, Via Università 100, 80055 Portici, Italy

e-mail: francesca.defilippis@unina.it; Phone: +39 081-2539388

ORCID: 0000-0002-3474-2884

Online Resource 1. NCBI genomes included in this study, with manually curated isolation sources.

L. gasseri genomes

Genome ID	Isolation source
GCF_000283135.1_ASM28313v1	Breast milk
GCF_013363915.1_ASM1336391v1	Breast milk
GCF_018728605.1_ASM1872860v1	Breast milk
GCF_022642475.1_ASM2264247v1	Human gut
GCF_018389265.1_ASM1838926v1	Human gut
GCF_925297925.1_IM1390	Human gut
GCF_019192945.1_ASM1919294v1	Breast milk
GCF_000014425.1_ASM1442v1	Human gut
GCF_001676665.1_ASM167666v1	Human gut
GCF_002003555.1_ASM200355v1	Human gut
GCF_002007185.1_ASM200718v1	Human gut
GCF_003307315.1_ASM330731v1	Human gut
GCF_003315575.1_ASM331557v1	Human gut
GCF_003437055.1_ASM343705v1	Human gut
GCF_005844525.1_ASM584452v1	Human gut
GCF_008868535.1_ASM886853v1	Human gut
GCF_015546835.1_ASM1554683v1	Human gut
GCF_015550315.1_ASM1555031v1	Human gut
GCF_017498665.1_ASM1749866v1	Human gut
GCF_017638885.1_ASM1763888v1	Human gut
GCF_017840575.1_ASM1784057v1	Human gut
GCF_024463435.1_ASM2446343v1	Human gut
GCF_027677045.1_ASM2767704v1	Human gut
GCF_027681405.1_ASM2768140v1	Human gut
GCF_027681485.1_ASM2768148v1	Human gut
GCF_027685305.1_ASM2768530v1	Human gut
GCF_902167745.1_Lactobacillus_gasseri_SV_Bg7063_mod2	Human gut
GCF_902386655.1_UHGG_MGYG-HGUT-02387	Human gut
GCF_902399865.1_UHGG_MGYG-HGUT-03690	Human gut
GCF_002158885.1_ASM215888v1	Infant feces
GCF_001063045.1_ASM106304v1	Human gut
GCF_001063065.1_ASM106306v1	Human gut
GCF_001063505.1_ASM106350v1	Human gut
GCF_001064985.1_ASM106498v1	Human gut
GCF_001066235.1_ASM106623v1	Human gut
GCF_001068345.1_ASM106834v1	Human gut
GCF_006981945.1_ASM698194v1	Not available

GCF_006982025.1_ASM698202v1	Not available
GCF_008868295.1_ASM886829v1	Not available
GCF_900452355.1_44087_F01	Human gut
GCF_020991185.1_ASM2099118v1	Human vagina
GCF_020991205.1_ASM2099120v1	Human vagina
GCF_020995385.1_ASM2099538v1	Human vagina
GCF_002287905.1_ASM228790v1	(human vagina) Vaginal capsules EcoVag
GCF_000175055.1_ASM17505v1	Human vagina
GCF_000176995.2_ASM17699v2	Human vagina
GCF_000177035.2_ASM17703v2	Human vagina
GCF_001546525.1_ASM154652v1	Human vagina
GCF_002863425.1_ASM286342v1	Human vagina
GCF_002863445.1_ASM286344v1	Human vagina
GCF_002863455.1_ASM286345v1	Human vagina
GCF_002884735.1_ASM288473v1	Human vagina
GCF_002940965.1_ASM294096v1	Human vagina
GCF_007785975.1_ASM778597v1	Human vagina
GCF_007785995.1_ASM778599v1	Human vagina
GCF_007786195.1_ASM778619v1	Human vagina
GCF_014654855.1_ASM1465485v1	Human vagina
GCF_017565825.1_ASM1756582v1	Human vagina
GCF_019277985.1_ASM1927798v1	Human vagina
GCF_022456925.1_ASM2245692v1	Human vagina
GCF_027584195.1_ASM2758419v1	Human vagina
GCF_000439915.2_ASM43991v2	Human vagina

***L. paragasseri* genomes**

**Genome ID**	**Isolation source**
GCF_000143645.1_ASM14364v1	Human vagina
GCF_000406345.2_LK71.0	Infant feces
GCF_003307255.1_ASM330725v1	Human gut
GCF_003307275.1_ASM330727v1	Human gut
GCF_003307295.1_ASM330729v1	Human gut
GCF_003584685.1_ASM358468v1	Human gut
GCF_005886075.1_ASM588607v1	Human vagina
GCF_007785845.1_ASM778584v1	Human vagina
GCF_007785885.1_ASM778588v1	Human vagina
GCF_007785945.1_ASM778594v1	Human vagina
GCF_009734365.1_ASM973436v1	Human gut
GCF_010092655.1_ASM1009265v1	Human vagina
GCF_014269295.2_ASM1426929v2	Human gut
GCF_015550525.1_ASM1555052v1	Human gut
GCF_015552765.1_ASM1555276v1	Human gut
GCF_015560405.1_ASM1556040v1	Human gut
GCF_018588245.1_ASM1858824v1	Human gut
GCF_019459585.1_ASM1945958v1	Human vagina
GCF_019972195.1_ASM1997219v1	Not available
GCF_022509305.1_ASM2250930v1	Fermented food
GCF_024972875.1_ASM2497287v1	Human vagina
GCF_024972895.1_ASM2497289v1	Human vagina
GCF_024972915.1_ASM2497291v1	Human vagina
GCF_025491465.1_ASM2549146v1	Human gut
GCF_027666495.1_ASM2766649v1	Human gut
GCF_027681345.1_ASM2768134v1	Human gut
GCF_028882125.1_ASM2888212v1	Human gut
GCF_028882155.1_ASM2888215v1	Human gut
GCF_028882165.1_ASM2888216v1	Human gut
GCF_900636895.1_44927_E01	Human vagina
GCF_925278945.1_IM216	Food additive
GCF_925299355.1_IM914	Breast milk

L. fermentum genomes

Genome ID	Isolation source
GCF_018884205.1_ASM1888420v1	Animal gut
GCF_023370755.1_ASM2337075v1	Animal gut
GCF_026184215.1_ASM2618421v1	Animal gut
GCF_025122575.1_ASM2512257v1	Fermented food
GCF_025191605.1_ASM2519160v1	Fermented food
GCF_025191705.1_ASM2519170v1	Fermented food
GCF_025191755.1_ASM2519175v1	Fermented food
GCF_009295755.1_ASM929575v1	Fermented food
GCF_020520555.1_ASM2052055v1	Breast milk
GCF_026930525.1_ASM2693052v1	Breast milk
GCF_024665195.1_ASM2466519v1	Commercial source (Chr. Hansen)
GCF_003053105.1_ASM305310v1	Commercial dietary supplements
GCF_003061865.1_ASM306186v1	Commercial dietary supplements
GCF_000397165.1_ASM39716v1	Fermented food
GCF_015160755.1_ASM1516075v1	Human gut
GCF_012273035.1_ASM1227303v1	Human gut
GCF_001368755.1_LF_newbler2.7	Fermented food
GCF_001982185.1_ASM198218v1	Fermented food
GCF_002242615.1_ASM224261v1	Fermented food
GCF_002356135.1_ASM235613v1	Fermented food
GCF_002798075.1_ASM279807v1	Fermented food
GCF_002899655.1_ASM289965v1	Fermented food
GCF_003261135.1_ASM326113v1	Fermented food
GCF_003346315.1_ASM334631v1	Fermented food
GCF_003346325.1_ASM334632v1	Fermented food
GCF_003346795.1_ASM334679v1	Fermented food
GCF_003855655.1_ASM385565v1	Fermented food
GCF_003860425.1_ASM386042v1	Fermented food
GCF_004683795.1_ASM468379v1	Fermented food
GCF_004683835.1_ASM468383v1	Fermented food
GCF_005341425.1_ASM534142v1	Fermented food
GCF_005864145.1_ASM586414v1	Fermented food
GCF_008802915.1_ASM880291v1	Fermented food
GCF_009362835.1_ASM936283v1	Fermented food
GCF_009676625.1_ASM967662v1	Fermented food
GCF_009914845.1_ASM991484v1	Fermented food
GCF_013394085.1_ASM1339408v1	Fermented food
GCF_016861955.1_ASM1686195v1	Fermented food
GCF_017134335.1_ASM1713433v1	Fermented food
GCF_017134355.1_ASM1713435v1	Fermented food
GCF_017134375.1_ASM1713437v1	Fermented food
GCF_018866265.1_ASM1886626v1	Fermented food

GCF_019890875.1_ASM1989087v1	Fermented food
GCF_020844625.1_ASM2084462v1	Fermented food
GCF_021128975.1_ASM2112897v1	Fermented food
GCF_021278125.1_ASM2127812v1	Fermented food
GCF_022509325.1_ASM2250932v1	Fermented food
GCF_022509335.1_ASM2250933v1	Fermented food
GCF_022509365.1_ASM2250936v1	Fermented food
GCF_022509385.1_ASM2250938v1	Fermented food
GCF_022509425.1_ASM2250942v1	Fermented food
GCF_022509445.1_ASM2250944v1	Fermented food
GCF_022509455.1_ASM2250945v1	Fermented food
GCF_022819245.1_ASM2281924v1	Fermented food
GCF_022844685.1_ASM2284468v1	Fermented food
GCF_023612195.1_ASM2361219v1	Fermented food
GCF_024800585.1_ASM2480058v1	Fermented food
GCF_025122475.1_ASM2512247v1	Fermented food
GCF_025185825.1_ASM2518582v1	Fermented food
GCF_025185835.1_ASM2518583v1	Fermented food
GCF_025185985.1_ASM2518598v1	Fermented food
GCF_025186015.1_ASM2518601v1	Fermented food
GCF_025191525.1_ASM2519152v1	Fermented food
GCF_025191625.1_ASM2519162v1	Fermented food
GCF_025191635.1_ASM2519163v1	Fermented food
GCF_025191785.1_ASM2519178v1	Fermented food
GCF_025191805.1_ASM2519180v1	Fermented food
GCF_025191825.1_ASM2519182v1	Fermented food
GCF_027857595.1_ASM2785759v1	Fermented food
GCF_027857625.1_ASM2785762v1	Fermented food
GCF_900163595.1_ASM90016359v1	Fermented food
GCF_900290185.1_L_fermentum_CECT_9269_Spades_Prokka	Fermented food
GCF_000010145.1_ASM1014v1	Fermented food
GCF_002204495.1_ASM220449v1	Fermented food
GCF_004063515.1_ASM406351v1	Fermented food
GCF_004063635.1_ASM406363v1	Fermented food
GCF_014926345.1_ASM1492634v1	Fermented food
GCF_016617695.1_ASM1661769v1	Fermented food
GCF_016861465.1_ASM1686146v1	Fermented food
GCF_016918895.1_ASM1691889v1	Fermented food
GCF_018732245.1_ASM1873224v1	Fermented food
GCF_021582915.1_ASM2158291v1	Fermented food
GCF_025191585.1_ASM2519158v1	Fermented food
GCF_025191645.1_ASM2519164v1	Fermented food
GCF_025191725.1_ASM2519172v1	Fermented food
GCF_025191745.1_ASM2519174v1	Fermented food
GCF_025211175.1_ASM2521117v1	Fermented food

GCF_025780195.1_ASM2578019v1	Fermented food
GCF_028462585.1_ASM2846258v1	Fermented food
GCF_028462685.1_ASM2846268v1	Fermented food
GCF_945190155.1_fsol_strainA	Animal (carcass)
GCF_945190205.1_fsol_strainC	Animal (carcass)
GCF_945190275.1_fsol_strainB	Animal (carcass)
GCF_014488995.1_ASM1448899v1	Breast milk
GCF_008868475.1_ASM886847v1	Human gut
GCF_001854105.1_ASM185410v1	Human gut
GCF_024137685.1_ASM2413768v1	Breast milk
GCF_000159215.1_ASM15921v1	Human gut
GCF_000417005.1_ASM41700v1	Human gut
GCF_000472265.1_LF1_1.0	Human gut
GCF_001039735.1_LFE2	Human gut
GCF_002119645.1_ASM211964v1	Human gut
GCF_002794275.1_ASM279427v1	Human gut
GCF_002794315.1_ASM279431v1	Human gut
GCF_002794375.1_ASM279437v1	Human gut
GCF_002869825.2_ASM286982v2	Human gut
GCF_003255875.1_ASM325587v1	Human gut
GCF_003462755.1_ASM346275v1	Human gut
GCF_003464285.1_ASM346428v1	Human gut
GCF_014830195.1_ASM1483019v1	Human gut
GCF_018398375.1_ASM1839837v1	Human gut
GCF_027661185.1_ASM2766118v1	Human gut
GCF_027668665.1_ASM2766866v1	Human gut
GCF_027681055.1_ASM2768105v1	Human gut
GCF_027681185.1_ASM2768118v1	Human gut
GCF_027681265.1_ASM2768126v1	Human gut
GCF_027686025.1_ASM2768602v1	Human gut
GCF_027696965.1_ASM2769696v1	Human gut
GCF_902363965.1_MGYG-HGUT-00166	Human gut
GCF_925284415.1_IM863	Breast milk
GCF_004208815.1_ASM420881v1	Infant faeces
GCF_011420355.1_ASM1142035v1	Infant faeces
GCF_012070605.1_ASM1207060v1	Infant faeces
GCF_012070625.1_ASM1207062v1	Infant faeces
GCF_001077025.1_ASM107702v1	Intensive care patient
GCF_000466785.3_ASM46678v3	Breast milk
GCF_001742205.1_ASM174220v1	Commercial source (Nestle)
GCF_000966835.2_ASM96683v2	Not available
GCF_001010185.1_ASM101018v1	Not available
GCF_001010245.1_ASM101024v1	Not available
GCF_001436835.1_ASM143683v1	Not available
GCF_006538825.1_ASM653882v1	Not available
GCF_024637935.1_ASM2463793v1	Not available

GCF_028463945.1_ASM2846394v1	Not available
GCF_028743095.1_ASM2874309v1	Not available
GCF_900163585.1_LFS6	Not available
GCF_002192435.1_ASM219243v1	Oral human cavity
GCF_002591935.1_ASM259193v1	Oral human cavity
GCF_011032745.1_ASM1103274v1	Oral human cavity
GCF_011032765.1_ASM1103276v1	Oral human cavity
GCF_011290755.1_ASM1129075v1	Oral human cavity
GCF_024054255.2_ASM2405425v2	Oral human cavity
GCF_024204625.1_ASM2420462v1	Oral human cavity
GCF_024385625.1_ASM2438562v1	Oral human cavity
GCF_025191505.1_ASM2519150v1	Oral human cavity
GCF_025191545.1_ASM2519154v1	Oral human cavity
GCF_025191565.1_ASM2519156v1	Oral human cavity
GCF_025809275.1_ASM2580927v1	Oral human cavity
GCF_028216035.1_ASM2821603v1	Oral human cavity
GCF_017068315.1_ASM1706831v1	probiotic supplement food
GCF_900205745.1_Lf_IMDO_130101	sourdough
GCF_000162395.1_ASM16239v1	Human vagina
GCF_000496435.1_LfermNB22_1.0	Human vagina
GCF_001941785.1_ASM194178v1	Human vagina
GCF_002863265.1_ASM286326v1	Human vagina
GCF_024494505.1_ASM2449450v1	Human vagina

***L. crispatus* genomes**

**Genome ID**	**Isolation source**
GCF_000091765.1_ASM9176v1	Animal gut
GCF_000160515.1_ASM16051v1	Human vagina
GCF_000161915.2_Lacto_cris_MV-1A-US_V2	Human vagina
GCF_000162255.1_ASM16225v1	Human vagina
GCF_000162315.1_ASM16231v1	Human vagina
GCF_000165885.1_ASM16588v1	Human vagina
GCF_000176975.2_ASM17697v2	Human vagina
GCF_000177575.1_ASM17757v1	Human vagina
GCF_000301115.1_Lact_cris_FB049-03_V1	Human vagina
GCF_000301135.1_Lact_cris_FB077-07_V1	Human vagina
GCF_000466885.3_ASM46688v3	Human vagina
GCF_000497065.1_L.crispatus1.0	Human gut
GCF_001434005.1_ASM143400v1	Human eye
GCF_001541385.1_ASM154138v1	Human vagina
GCF_001541405.1_ASM154140v1	Human vagina
GCF_001541505.1_ASM154150v1	Human vagina
GCF_001541515.1_ASM154151v1	Human vagina
GCF_001541535.1_ASM154153v1	Human vagina
GCF_001541585.1_ASM154158v1	Human vagina
GCF_001546015.1_ASM154601v1	Human vagina
GCF_001546025.1_ASM154602v1	Human vagina
GCF_001563615.1_ASM156361v1	Human urine
GCF_001567095.1_ASM156709v1	Animal gut
GCF_001700475.1_ASM170047v1	Human urine
GCF_001704465.1_ASM170446v1	Animal gut
GCF_002088015.1_ASM208801v1	Not available
GCF_002218565.1_ASM221856v1	Animal gut
GCF_002218615.1_ASM221861v1	Animal gut
GCF_002218645.1_ASM221864v1	Animal gut
GCF_002218655.1_ASM221865v1	Animal gut
GCF_002218685.1_ASM221868v1	Animal gut
GCF_002218695.1_ASM221869v1	Animal gut
GCF_002218735.1_ASM221873v1	Animal gut
GCF_002218765.1_ASM221876v1	Animal gut
GCF_002218775.1_ASM221877v1	Animal gut
GCF_002218805.1_ASM221880v1	Animal gut
GCF_002218815.1_ASM221881v1	Animal gut
GCF_002218845.1_ASM221884v1	Animal gut
GCF_002218855.1_ASM221885v1	Animal gut
GCF_002218885.1_ASM221888v1	Animal gut

GCF_002218895.1_ASM221889v1	Animal gut
GCF_002218925.1_ASM221892v1	Animal gut
GCF_002218945.1_ASM221894v1	Animal gut
GCF_002218965.1_ASM221896v1	Animal gut
GCF_002218975.1_ASM221897v1	Animal gut
GCF_002219005.1_ASM221900v1	Animal gut
GCF_002219015.1_ASM221901v1	Animal gut
GCF_002219045.1_ASM221904v1	Animal gut
GCF_002219055.1_ASM221905v1	Animal gut
GCF_002219085.1_ASM221908v1	Animal gut
GCF_002811165.1_ASM281116v1	Oral human cavity
GCF_002861765.1_ASM286176v1	Human vagina
GCF_002861775.1_ASM286177v1	Human vagina
GCF_002861805.1_ASM286180v1	Human vagina
GCF_002861815.1_ASM286181v1	Human vagina
GCF_002863245.1_ASM286324v1	Human vagina
GCF_002863485.1_ASM286348v1	Human vagina
GCF_002863505.1_ASM286350v1	Human vagina
GCF_003795065.1_ASM379506v1	Human vagina
GCF_003971565.1_ASM397156v1	Human vagina
GCF_004103355.1_ASM410335v1	Human vagina
GCF_004334905.1_ASM433490v1	Animal gut
GCF_004361075.1_ASM436107v1	Human vagina
GCF_004361095.1_ASM436109v1	Human vagina
GCF_004361115.1_ASM436111v1	Human vagina
GCF_004361125.1_ASM436112v1	Human vagina
GCF_004361175.1_ASM436117v1	Human vagina
GCF_004361185.1_ASM436118v1	Human vagina
GCF_004361195.1_ASM436119v1	Human vagina
GCF_004361205.1_ASM436120v1	Human vagina
GCF_004361215.1_ASM436121v1	Human vagina
GCF_004361245.1_ASM436124v1	Human vagina
GCF_004361265.1_ASM436126v1	Human vagina
GCF_004361295.1_ASM436129v1	Human vagina
GCF_004361315.1_ASM436131v1	Human vagina
GCF_004361345.1_ASM436134v1	Human vagina
GCF_004361355.1_ASM436135v1	Human vagina
GCF_004361375.1_ASM436137v1	Human vagina
GCF_004361385.1_ASM436138v1	Human vagina
GCF_004361395.1_ASM436139v1	Human vagina
GCF_004361445.1_ASM436144v1	Human vagina
GCF_004361455.1_ASM436145v1	Human vagina
GCF_004361465.1_ASM436146v1	Human vagina

GCF_004361475.1_ASM436147v1	Human vagina
GCF_004361515.1_ASM436151v1	Human vagina
GCF_004361545.1_ASM436154v1	Human vagina
GCF_004361555.1_ASM436155v1	Human vagina
GCF_004361565.1_ASM436156v1	Human vagina
GCF_004361575.1_ASM436157v1	Human vagina
GCF_004361635.1_ASM436163v1	Human vagina
GCF_004681235.1_ASM468123v1	Human vagina
GCF_007713895.1_ASM771389v1	Human gut
GCF_008079315.1_ASM807931v1	Human vagina
GCF_008694205.1_ASM869420v1	Animal gut
GCF_008694745.1_ASM869474v1	Not available
GCF_008694755.1_ASM869475v1	Human eye
GCF_008694765.1_ASM869476v1	Animal gut
GCF_008694775.1_ASM869477v1	Animal gut
GCF_008694785.1_ASM869478v1	Animal gut
GCF_008694845.1_ASM869484v1	Animal gut
GCF_008694865.1_ASM869486v1	Animal gut
GCF_008694875.1_ASM869487v1	Animal gut
GCF_008694885.1_ASM869488v1	Animal gut
GCF_008694925.1_ASM869492v1	Animal gut
GCF_008694935.1_ASM869493v1	Animal gut
GCF_008694975.1_ASM869497v1	Animal gut
GCF_008694985.1_ASM869498v1	Not available
GCF_008868575.1_ASM886857v1	Human vagina
GCF_009730275.1_ASM973027v1	Human vagina
GCF_009769205.1_ASM976920v1	Animal gut
GCF_009857225.1_ASM985722v1	Human vagina
GCF_009857395.1_ASM985739v1	Human vagina
GCF_009933525.1_ASM993352v1	Animal gut
GCF_011029265.1_ASM1102926v1	Human vagina
GCF_012030075.1_ASM1203007v1	Human vagina
GCF_012843585.1_ASM1284358v1	Animal gut
GCF_013456995.1_ASM1345699v1	Human gut
GCF_013487905.1_ASM1348790v1	Animal gut
GCF_013778545.1_ASM1377854v1	Fermented food
GCF_014654865.1_Biofab_BC5_1.1	Human vagina
GCF_014982905.1_ASM1498290v1	Animal gut
GCF_015669875.1_ASM1566987v1	Human gut
GCF_015708055.1_ASM1570805v1	Human vagina
GCF_015708065.1_ASM1570806v1	Human vagina
GCF_015708075.1_ASM1570807v1	Human vagina
GCF_015708105.1_ASM1570810v1	Human vagina

GCF_016093195.1_ASM1609319v1	Human vagina
GCF_016161925.1_ASM1616192v1	Animal gut
GCF_016161975.1_ASM1616197v1	Animal gut
GCF_016161995.1_ASM1616199v1	Animal gut
GCF_016162005.1_ASM1616200v1	Animal gut
GCF_016162045.1_ASM1616204v1	Animal gut
GCF_016162055.1_ASM1616205v1	Animal gut
GCF_016162065.1_ASM1616206v1	Human vagina
GCF_016162105.1_ASM1616210v1	Animal gut
GCF_016162125.1_ASM1616212v1	Human vagina
GCF_016162145.1_ASM1616214v1	Human vagina
GCF_016162155.1_ASM1616215v1	Human vagina
GCF_016162165.1_ASM1616216v1	Human vagina
GCF_016162185.1_ASM1616218v1	Human vagina
GCF_016162195.1_ASM1616219v1	Human vagina
GCF_016767795.1_ASM1676779v1	Human vagina
GCF_016901535.1_ASM1690153v1	Animal gut
GCF_017599225.1_ASM1759922v1	Human gut
GCF_018885325.1_ASM1888532v1	Human vagina
GCF_018987235.1_ASM1898723v1	Oral human cavity
GCF_019277925.1_ASM1927792v1	Human vagina
GCF_019278055.1_ASM1927805v1	Human vagina
GCF_019278075.1_ASM1927807v1	Human vagina
GCF_019278105.1_ASM1927810v1	Human vagina
GCF_019278135.1_ASM1927813v1	Human vagina
GCF_019537355.1_ASM1953735v1	Human vagina
GCF_020042005.1_ASM2004200v1	Human vagina
GCF_020042125.1_ASM2004212v1	Human vagina
GCF_020042225.1_ASM2004222v1	Human vagina
GCF_020224235.1_ASM2022423v1	Human gut
GCF_020887095.1_ASM2088709v1	Human gut
GCF_021278925.1_ASM2127892v1	Human vagina
GCF_021278945.1_ASM2127894v1	Human vagina
GCF_022453955.1_ASM2245395v1	Human vagina
GCF_022453975.1_ASM2245397v1	Human vagina
GCF_022454015.1_ASM2245401v1	Human vagina
GCF_022454025.1_ASM2245402v1	Human vagina
GCF_022454055.1_ASM2245405v1	Human vagina
GCF_022454075.1_ASM2245407v1	Human vagina
GCF_022454115.1_ASM2245411v1	Human vagina
GCF_022454135.1_ASM2245413v1	Human vagina
GCF_022454155.1_ASM2245415v1	Human vagina
GCF_022454175.1_ASM2245417v1	Human vagina

GCF_022454205.1_ASM2245420v1	Human vagina
GCF_022454275.1_ASM2245427v1	Human vagina
GCF_022454285.1_ASM2245428v1	Human vagina
GCF_022454315.1_ASM2245431v1	Human vagina
GCF_022454335.1_ASM2245433v1	Human vagina
GCF_022454355.1_ASM2245435v1	Human vagina
GCF_022454375.1_ASM2245437v1	Human vagina
GCF_022454385.1_ASM2245438v1	Human vagina
GCF_022454395.1_ASM2245439v1	Human vagina
GCF_022454435.1_ASM2245443v1	Human vagina
GCF_022454475.1_ASM2245447v1	Human vagina
GCF_022454495.1_ASM2245449v1	Human vagina
GCF_022454515.1_ASM2245451v1	Human vagina
GCF_022454535.1_ASM2245453v1	Human vagina
GCF_022454555.1_ASM2245455v1	Human vagina
GCF_022454565.1_ASM2245456v1	Human vagina
GCF_022454615.1_ASM2245461v1	Human vagina
GCF_022454655.1_ASM2245465v1	Human vagina
GCF_022454675.1_ASM2245467v1	Human vagina
GCF_022454695.1_ASM2245469v1	Human vagina
GCF_022454705.1_ASM2245470v1	Human vagina
GCF_022454735.1_ASM2245473v1	Human vagina
GCF_022454755.1_ASM2245475v1	Human vagina
GCF_022454765.1_ASM2245476v1	Human vagina
GCF_022454795.1_ASM2245479v1	Human vagina
GCF_022454815.1_ASM2245481v1	Human vagina
GCF_022454835.1_ASM2245483v1	Human vagina
GCF_022454845.1_ASM2245484v1	Human vagina
GCF_022454865.1_ASM2245486v1	Human vagina
GCF_022454895.1_ASM2245489v1	Human vagina
GCF_022454915.1_ASM2245491v1	Human vagina
GCF_022454935.1_ASM2245493v1	Human vagina
GCF_022454955.1_ASM2245495v1	Human vagina
GCF_022454965.1_ASM2245496v1	Human vagina
GCF_022455005.1_ASM2245500v1	Human vagina
GCF_022455035.1_ASM2245503v1	Human vagina
GCF_022455055.1_ASM2245505v1	Human vagina
GCF_022455065.1_ASM2245506v1	Human vagina
GCF_022455115.1_ASM2245511v1	Human vagina
GCF_022455135.1_ASM2245513v1	Human vagina
GCF_022455155.1_ASM2245515v1	Human vagina
GCF_022455175.1_ASM2245517v1	Human vagina
GCF_022455185.1_ASM2245518v1	Human vagina

GCF_022455205.1_ASM2245520v1	Human vagina
GCF_022455235.1_ASM2245523v1	Human vagina
GCF_022455255.1_ASM2245525v1	Human vagina
GCF_022455265.1_ASM2245526v1	Human vagina
GCF_022455295.1_ASM2245529v1	Human vagina
GCF_022455315.1_ASM2245531v1	Human vagina
GCF_022455325.1_ASM2245532v1	Human vagina
GCF_022455355.1_ASM2245535v1	Human vagina
GCF_022455375.1_ASM2245537v1	Human vagina
GCF_022455395.1_ASM2245539v1	Human vagina
GCF_022455415.1_ASM2245541v1	Human vagina
GCF_022455455.1_ASM2245545v1	Human vagina
GCF_022455475.1_ASM2245547v1	Human vagina
GCF_022455485.1_ASM2245548v1	Human vagina
GCF_022455515.1_ASM2245551v1	Human vagina
GCF_022455535.1_ASM2245553v1	Human vagina
GCF_022455555.1_ASM2245555v1	Human vagina
GCF_022455575.1_ASM2245557v1	Human vagina
GCF_022455595.1_ASM2245559v1	Human vagina
GCF_022455615.1_ASM2245561v1	Human vagina
GCF_022455635.1_ASM2245563v1	Human vagina
GCF_022455655.1_ASM2245565v1	Human vagina
GCF_022455675.1_ASM2245567v1	Human vagina
GCF_022455695.1_ASM2245569v1	Human vagina
GCF_022455705.1_ASM2245570v1	Human vagina
GCF_022455735.1_ASM2245573v1	Human vagina
GCF_022455755.1_ASM2245575v1	Human vagina
GCF_022455855.1_ASM2245585v1	Human vagina
GCF_022455875.1_ASM2245587v1	Human vagina
GCF_022455895.1_ASM2245589v1	Human vagina
GCF_022455915.1_ASM2245591v1	Human vagina
GCF_022455935.1_ASM2245593v1	Human vagina
GCF_022455955.1_ASM2245595v1	Human vagina
GCF_022455975.1_ASM2245597v1	Human vagina
GCF_022455985.1_ASM2245598v1	Human vagina
GCF_022456015.1_ASM2245601v1	Human vagina
GCF_022456035.1_ASM2245603v1	Human vagina
GCF_022456175.1_ASM2245617v1	Human vagina
GCF_022456215.1_ASM2245621v1	Human vagina
GCF_022456295.1_ASM2245629v1	Human vagina
GCF_022456575.1_ASM2245657v1	Human vagina
GCF_022456595.1_ASM2245659v1	Human vagina
GCF_022456605.1_ASM2245660v1	Human vagina

GCF_022456655.1_ASM2245665v1	Human vagina
GCF_022456695.1_ASM2245669v1	Human vagina
GCF_022456705.1_ASM2245670v1	Human vagina
GCF_022456735.1_ASM2245673v1	Human vagina
GCF_022456755.1_ASM2245675v1	Human vagina
GCF_022456775.1_ASM2245677v1	Human vagina
GCF_022456795.1_ASM2245679v1	Human vagina
GCF_022456805.1_ASM2245680v1	Human vagina
GCF_022456835.1_ASM2245683v1	Human vagina
GCF_022456855.1_ASM2245685v1	Human vagina
GCF_022456875.1_ASM2245687v1	Human vagina
GCF_022456895.1_ASM2245689v1	Human vagina
GCF_022456935.1_ASM2245693v1	Human vagina
GCF_022456975.1_ASM2245697v1	Human vagina
GCF_022456995.1_ASM2245699v1	Human vagina
GCF_022457405.1_ASM2245740v1	Human vagina
GCF_022457425.1_ASM2245742v1	Human vagina
GCF_022457495.1_ASM2245749v1	Human vagina
GCF_022457545.1_ASM2245754v1	Human vagina
GCF_022458295.1_ASM2245829v1	Human vagina
GCF_022458335.1_ASM2245833v1	Human vagina
GCF_022458415.1_ASM2245841v1	Human vagina
GCF_022458425.1_ASM2245842v1	Human vagina
GCF_022458495.1_ASM2245849v1	Human vagina
GCF_025194045.1_ASM2519404v1	Not available
GCF_025194085.1_ASM2519408v1	Infant gut
GCF_025194105.1_ASM2519410v1	Not available
GCF_025194115.1_ASM2519411v1	Animal gut
GCF_026740115.1_ASM2674011v1	Infant gut
GCF_027152805.1_ASM2715280v1	Human vagina
GCF_027152825.1_ASM2715282v1	Human vagina
GCF_027152845.1_ASM2715284v1	Human vagina
GCF_027152885.1_ASM2715288v1	Human vagina
GCF_027153255.1_ASM2715325v1	Human vagina
GCF_027153305.1_ASM2715330v1	Human vagina
GCF_027153325.1_ASM2715332v1	Human vagina
GCF_027153355.1_ASM2715335v1	Human vagina
GCF_027153385.1_ASM2715338v1	Human vagina
GCF_027153425.1_ASM2715342v1	Human vagina
GCF_027153445.1_ASM2715344v1	Human vagina
GCF_027153465.1_ASM2715346v1	Human vagina
GCF_027153485.1_ASM2715348v1	Human vagina
GCF_027153505.1_ASM2715350v1	Human vagina

GCF_027153525.1_ASM2715352v1	Human vagina
GCF_027153545.1_ASM2715354v1	Human vagina
GCF_027153565.1_ASM2715356v1	Human vagina
GCF_027153585.1_ASM2715358v1	Human vagina
GCF_027153605.1_ASM2715360v1	Human vagina
GCF_027153625.1_ASM2715362v1	Human vagina
GCF_027153645.1_ASM2715364v1	Human vagina
GCF_027153665.1_ASM2715366v1	Human vagina
GCF_027153685.1_ASM2715368v1	Human vagina
GCF_027153705.1_ASM2715370v1	Human vagina
GCF_027153715.1_ASM2715371v1	Human vagina
GCF_027153745.1_ASM2715374v1	Human vagina
GCF_027153755.1_ASM2715375v1	Human vagina
GCF_027153785.1_ASM2715378v1	Human vagina
GCF_027153805.1_ASM2715380v1	Human vagina
GCF_027153815.1_ASM2715381v1	Human vagina
GCF_027153845.1_ASM2715384v1	Human vagina
GCF_027153925.1_ASM2715392v1	Human vagina
GCF_027153945.1_ASM2715394v1	Human vagina
GCF_027153965.1_ASM2715396v1	Human vagina
GCF_027153985.1_ASM2715398v1	Human vagina
GCF_027154005.1_ASM2715400v1	Human vagina
GCF_027154025.1_ASM2715402v1	Human vagina
GCF_027154035.1_ASM2715403v1	Human vagina
GCF_027154065.1_ASM2715406v1	Human vagina
GCF_027154085.1_ASM2715408v1	Human vagina
GCF_027154125.1_ASM2715412v1	Human vagina
GCF_027154135.1_ASM2715413v1	Human vagina
GCF_027154285.1_ASM2715428v1	Human vagina
GCF_027154295.1_ASM2715429v1	Human vagina
GCF_027155005.1_ASM2715500v1	Human vagina
GCF_027155045.1_ASM2715504v1	Human vagina
GCF_027155075.1_ASM2715507v1	Human vagina
GCF_027155105.1_ASM2715510v1	Human vagina
GCF_027155375.1_ASM2715537v1	Human vagina
GCF_027155425.1_ASM2715542v1	Human vagina
GCF_027155435.1_ASM2715543v1	Human vagina
GCF_027155545.1_ASM2715554v1	Human vagina
GCF_027155575.1_ASM2715557v1	Human vagina
GCF_027156705.1_ASM2715670v1	Human vagina
GCF_027156725.1_ASM2715672v1	Human vagina
GCF_027157565.1_ASM2715756v1	Human vagina
GCF_027157665.1_ASM2715766v1	Human vagina

GCF_027158685.1_ASM2715868v1	Human vagina
GCF_027158705.1_ASM2715870v1	Human vagina
GCF_027158725.1_ASM2715872v1	Human vagina
GCF_027158735.1_ASM2715873v1	Human vagina
GCF_027158765.1_ASM2715876v1	Human vagina
GCF_027158785.1_ASM2715878v1	Human vagina
GCF_027158795.1_ASM2715879v1	Human vagina
GCF_027158825.1_ASM2715882v1	Human vagina
GCF_027158835.1_ASM2715883v1	Human vagina
GCF_027158845.1_ASM2715884v1	Human vagina
GCF_027158885.1_ASM2715888v1	Human vagina
GCF_027158905.1_ASM2715890v1	Human vagina
GCF_027158925.1_ASM2715892v1	Human vagina
GCF_027158945.1_ASM2715894v1	Human vagina
GCF_027158985.1_ASM2715898v1	Human vagina
GCF_027159005.1_ASM2715900v1	Human vagina
GCF_027159025.1_ASM2715902v1	Human vagina
GCF_027159035.1_ASM2715903v1	Human vagina
GCF_027159065.1_ASM2715906v1	Human vagina
GCF_027159085.1_ASM2715908v1	Human vagina
GCF_027159095.1_ASM2715909v1	Human vagina
GCF_027160085.1_ASM2716008v1	Human vagina
GCF_027160095.1_ASM2716009v1	Human vagina
GCF_027160165.1_ASM2716016v1	Human vagina
GCF_027160215.1_ASM2716021v1	Human vagina
GCF_027160225.1_ASM2716022v1	Human vagina
GCF_027160285.1_ASM2716028v1	Human vagina
GCF_027160325.1_ASM2716032v1	Human vagina
GCF_027160525.1_ASM2716052v1	Human vagina
GCF_027160545.1_ASM2716054v1	Human vagina
GCF_027160555.1_ASM2716055v1	Human vagina
GCF_027160585.1_ASM2716058v1	Human vagina
GCF_027160595.1_ASM2716059v1	Human vagina
GCF_027160805.1_ASM2716080v1	Human vagina
GCF_027160825.1_ASM2716082v1	Human vagina
GCF_027160845.1_ASM2716084v1	Human vagina
GCF_027160865.1_ASM2716086v1	Human vagina
GCF_027161085.1_ASM2716108v1	Human vagina
GCF_027161105.1_ASM2716110v1	Human vagina
GCF_027161115.1_ASM2716111v1	Human vagina
GCF_027161125.1_ASM2716112v1	Human vagina
GCF_027161165.1_ASM2716116v1	Human vagina
GCF_027271175.1_ASM2727117v1	Human vagina

GCF_027583895.1_ASM2758389v1	Human vagina
GCF_027583915.1_ASM2758391v1	Human vagina
GCF_027583955.1_ASM2758395v1	Human vagina
GCF_027583975.1_ASM2758397v1	Human vagina
GCF_027583995.1_ASM2758399v1	Human vagina
GCF_027584035.1_ASM2758403v1	Human vagina
GCF_027584095.1_ASM2758409v1	Human vagina
GCF_027584215.1_ASM2758421v1	Human vagina
GCF_027680585.1_ASM2768058v1	Human gut
GCF_027682305.1_ASM2768230v1	Human gut
GCF_027695305.1_ASM2769530v1	Human gut
GCF_902386155.1_UHGG_MGYG-HGUT-02348	Human gut
