# Supplementary material for: Women’s health is a team effort: probiogenomics supports the development of a multi-species vaginal probiotic
Source: Cell Mol Life Sci. 2026 Feb 26;83(1):132. doi: 10.1007/s00018-026-06107-2 (PMC12957687; doi:10.1007/s00018-026-06107-2)
Supplement: Supplementary file 2 — Supplementary Material 2 (PDF 249 KB) [file 18_2026_6107_MOESM2_ESM.pdf]

Women's health is a team effort: probiogenomics supports the development of a multi-species vaginal probiotic

Chiara Maria Calvanese[1], Vincenzo Valentino[1], Annachiara De Prisco[2], Serena Allesina[2], Angela Amoruso[2], Francesca Deidda[2], Annalisa Visciglia[2], Danilo Ercolini[1,3], Marco Pane[2], Francesca De Filippis[1,3*]

[1] Department of Agricultural Sciences, University of Naples Federico II, P.zza Carlo di Borbone 1, 80055 Portici (NA), Italy

[2]Probiotical Research S.r.l., via Enrico Mattei 3, 28100 Novara, Italy

[3] Task Force on Microbiome Studies, University of Naples Federico II, Corso Umberto I 43, 80100 Napoli, Italy

Journal: Cellular and Molecular Life Sciences

Corresponding Author:

Prof. Francesca De Filippis

Department of Agricultural Sciences, University of Naples Federico II, Via Università 100, 80055 Portici, Italy

e-mail: francesca.defilippis@unina.it; Phone: +39 081-2539388

ORCID: 0000-0002-3474-2884

Online Resource 2. Genes included in the custom database and NCBI accessions.

Vaginal adaption genes

Group	NCBI accession number	Annotation	Organism	Reference
Adhesion and Colonization	pdb|7BVX|A	SpaCBA	*Lacticaseibacillus rhamnosus*	(F. Zhang et al., 2021)
Adhesion and Colonization	pdb|6M48|B	MSCRAMM family adhesin SdrC	*Lacticaseibacillus rhamnosus*	(F. Zhang et al., 2021)
Adhesion and Colonization	SPS13880.1	Collagen adhesin can	*Lactobacillus helveticus*	(F. Zhang et al., 2021)
Adhesion and Colonization	AAY41912.1	S-layer protein SlpA	*Lactobacillus crispatus*	(Sanozky-Dawes & Barrangou, 2022)
Adhesion and Colonization	AAY41916.1	S-layer protein SlpB	*Lactobacillus crispatus*	(Sanozky-Dawes & Barrangou, 2022)
Adhesion and Colonization	KAA8781809.1	S-layer protein SlpX	*Lactobacillus crispatus*	(Sanozky-Dawes & Barrangou, 2022)
Adhesion and Colonization	WP_270223762.1	Beta-N-acetylglucosaminidase AcmB S-layer	*Lactobacillus acidophilus*	(Sanozky-Dawes & Barrangou, 2022)
Adhesion and Colonization	AGT42024.1	Lectin-like protein 1	*Lacticaseibacillus rhamnosus*	(Petrova et al., 2016)
Adhesion and Colonization	WP_014569179.1	Lectin-like protein 2	*Lacticaseibacillus rhamnosus*	(Petrova et al., 2016)
Adhesion and Colonization	WP_239665380.1	Adhesin	*Lactobacillus*	(Dertli et al., 2016; Lebeer et al., 2008)
Antimicrobial Production and Microbial Interactions	WP_225907783.1	Lantibiotic dehydratase	*Lactobacillus crispatus*	(Darvishi et al., 2021)
Antimicrobial Production and Microbial Interactions	AAA88606.1	Nisin	*Lactococcus lactis*	(Darvishi et al., 2021)
Antimicrobial Production and	CAA87640.1	Mersacidin	*Bacillus sp.*	(Darvishi et al., 2021)

Microbial Interactions				
Antimicrobial Production and Microbial Interactions	QHM51289.1	Lysostaphin	*Lactiplantibacillus plantarum*	(Darvishi et al., 2021)
Antimicrobial Production and Microbial Interactions	WP_112324691.1	Pesticin	*Enterobacteriaceae*	(Darvishi et al., 2021)
Antimicrobial Production and Microbial Interactions	WP_220269064.1	Colicin	*Lactobacillus*	(Darvishi et al., 2021)
Antimicrobial Production and Microbial Interactions	WP_010890686.1	Lantibiotic lacticin	*Bacilli*	(Darvishi et al., 2021)
Antimicrobial Production and Microbial Interactions	WP_011005836.1	Lactocin 705 family bacteriocin	*Latilactobacillus*	(Darvishi et al., 2021)
Antimicrobial Production and Microbial Interactions	WP_198183798.1	Helveticin J family class III bacteriocin	*Lactobacillus*	(Darvishi et al., 2021)
Antimicrobial Production and Microbial Interactions	WP_278229227.1	lanthionine synthetase LanC family protein	*Lactococcus lactis*	(Perez et al., 2022)
Antimicrobial Production and Microbial Interactions	VTU64604.1	Pediocin PapC-like protein	*Lacticaseibacillus rhamnosus*	(Perez et al., 2022)
Antimicrobial Production and Microbial Interactions	WP_089144005.1	enterocin L50 family leaderless bacteriocin	*Bacilli*	(Perez et al., 2022)
Antimicrobial Production and Microbial Interactions	ALJ02606.1	Plantaricin	*Lactiplantibacillus plantarum*	(Perez et al., 2022)
Antimicrobial Production and Microbial Interactions	pdb|1OHN|A	Sakacin chain A	*Latilactobacillus sakei*	(Perez et al., 2022)
Carbohydrate metabolism in the vaginal environment	CAX67156.1	inulosucrase InuJ	*Lactobacillus johnsonii*	(J. Zhang et al., 2022)

Carbohydrate metabolism in the vaginal environment	RXF55115.1	Levansucrase (InuCA e InRA)	*Lactobacillus crispatus*	(J. Zhang et al., 2022)
Carbohydrate metabolism in the vaginal environment	KAA9244007.1	Sialidase	*Lactobacillus mulieris*	(Ferreira et al., 2022)
Carbohydrate metabolism in the vaginal environment	GMB71175.1	Amylopullulanase	*Lacticaseibacillus rhamnosus*	(Hertzberger et al., 2022)
Carbohydrate metabolism in the vaginal environment	WP_052542819.1	Type I pullulanase pulA	*Lactobacillus*	(Hertzberger et al., 2022)
Carbohydrate metabolism in the vaginal environment	BBD47863.1	Alpha-amylase	*Lactobacillus paragasseri*	(Nunn et al., 2020)
Carbohydrate metabolism in the vaginal environment	KRK35887.1	Glucan 1,6-alpha-glucosidase	*Lactobacillus crispatus*	(Nunn et al., 2020)
Carbohydrate metabolism in the vaginal environment	GBA94374.1	Oligo-1,6-glucosidase	*Lactobacillus gasseri*	(Nunn et al., 2020)
Carbohydrate metabolism in the vaginal environment	EEU19064.1	Intracellular maltogenic amylase	*Lactobacillus crispatus*	(Nunn et al., 2020)
Carbohydrate metabolism in the vaginal environment	CAA0276191.1	Trehalose synthase/amylase TreS	*Klebsiella oxytoca*	(Nunn et al., 2020)
Carbohydrate metabolism in the vaginal environment	CAH9055892.1	Glycogen operon protein GlgX	*Pseudoalteromonas haloplanktis*	(Nunn et al., 2020)
Carbohydrate metabolism in the vaginal environment	KWU04919.1	Pullulanase	*Lactobacillus crispatus*	(Nunn et al., 2020)
Carbohydrate metabolism in the vaginal environment	KRK32829.1	Neopullulanase	*Lactobacillus crispatus*	(Nunn et al., 2020)
Carbohydrate metabolism in	WP_013439216.1	UTP--glucose-1-phosphate	*Lactobacillus*	(F. Zhang et al., 2021)

the vaginal environment				
Carbohydrate metabolism in the vaginal environment	CBJ19544.1	Glucosyltransferase gtf	*Lentilactobacillus hilgardii*	(Dertli et al., 2016; Lebeer et al., 2008)
Degradation of biogenic amines	EEL71807.1	Putrescine aminotransferase	*Bacillus mycoides*	(Guarcello et al., 2016; Puebla-Barragan et al., 2021)
Host Interaction and Immunomodulation	WP_198194524.1	SLAP domain-containing protein	*Lactobacillus*	(Sanozky-Dawes & Barrangou, 2022)
Host Interaction and Immunomodulation	WP_198182809.1	UDP-GlcNAc/MurNAc family	*Lactobacillus*	(Chee et al., 2020)
Host Interaction and Immunomodulation	WP_089147280.1	LTA synthase family protein	*Lactobacillus*	(Chee et al., 2020)
Host Interaction and Immunomodulation	UQD53807.1	Biosurfactants production protein	*Bacillus methanolicus*	(Chee et al., 2020)
Host Interaction and Immunomodulation	WP_005687638.1	Peptidoglycan hydrolase Msp1	*Lacticaseibacillus rhamnosus*	(Chee et al., 2020)
Host Interaction and Immunomodulation	CUS16278.1	S-ribosylhomocysteine lyase / quorum-sensing autoinducer 2 (AI-2) synthesis protein LuxS	*Lactobacillus delbrueckii subsp. Bulgaricus*	(Dertli et al., 2016; Lebeer et al., 2008)
Production of biogenic amines	WP_002319546.1	Tyrosine decarboxylase	*Enterococcus*	(Guarcello et al., 2016)
Production of biogenic amines	CBX24604.1	Histidine decarboxylase	*Streptococcus thermophilus*	(Guarcello et al., 2016)
Production of biogenic amines	WP_001292415.1	Ornithine decarboxylase	*Escherichia*	(Guarcello et al., 2016)
Production of biogenic amines	WP_001295383.1	Lysine decarboxylase	*Enterobacteriaceae*	(Guarcello et al., 2016)
Production of biogenic amines	WP_009911777.1	Agmatine deiminase	*Listeria monocytogenes*	(Guarcello et al., 2016)
Production of biogenic amines	WP_001381593.1	Arginine decarboxylase	*Enterobacteriaceae*	(Guarcello et al., 2016)
Production of biogenic amines	BAM13386.1	Arginine racemase	*Pseudomonas taetrolens*	(Guarcello et al., 2016)

Production of biogenic amines	BBG56304.1	Lysine racemase	*Leuconostoc mesenteroides*	(Guarcello et al., 2016; Puebla-Barragan et al., 2021)
Stress Response and Survival	WP_001189647.1	Multicopper oxidase	*Enterobacteriaceae*	(Guarcello et al., 2016; Puebla-Barragan et al., 2021)
Stress Response and Survival	ABM21381.1	Eps transcriptional regulator	*Lactobacillus johnsonii*	(Dertli et al., 2016; Lebeer et al., 2008)
Stress Response and Survival	WP_394857538.1	MMPL transporter FarE	*Staphylococcus aureus*	(Zhu et al. 2024)
Stress Response and Survival	gi|489752033|ref|WP_003656035.1|	MMPL family transporter	*Lactobacillus sp.*	(Zhu et al. 2024)
Stress Response and Survival	SNX31820.1	oleate hydratase	*Limosilactobacillus fermentum*	(Zhu et al. 2024)

Probiotic factor genes

Functional group	NCBI accession number	Annotation	Reference
Mechanisms of active response to stress related to GIT passage	AAD01782.1	ClpE [*Lactococcus cremoris subsp. cremoris* MG1363]	Leeber et al., 2008
Mechanisms of active response to stress related to GIT passage	AAG44707.1	EpsC [*Lactobacillus delbrueckii subsp. bulgaricus*]	Leeber et al., 2008
Mechanisms of active response to stress related to GIT passage	AAG44708.1	EpsD [*Lactobacillus delbrueckii subsp. bulgaricus*]	Leeber et al., 2008
Mechanisms of active response to stress related to GIT passage	AAG44709.1	phospho-glucosyltransferase EpsE [*Lactobacillus delbrueckii subsp. bulgaricus*]	Leeber et al., 2008
Mechanisms of active response to stress related to GIT passage	AAG44712.1	beta(1,3)galactosyltransferase EpsH [*Lactobacillus delbrueckii subsp. bulgaricus*]	Leeber et al., 2008
Mechanisms of active response to stress related to GIT passage	AAK64290.2	ClpL [*Lacticaseibacillus rhamnosus*]	Leeber et al., 2008
Mechanisms of active response to stress related to GIT passage	AAS08038.1	conjugated bile salt hydrolase [*Lactobacillus johnsonii* NCC 533]	Leeber et al., 2008
Mechanisms of active response to stress related to GIT passage	AAS08969.1	conjugated bile salt hydrolase [*Lactobacillus johnsonii* NCC 533]	Leeber et al., 2008
Mechanisms of active response to stress related to GIT passage	AAS09179.1	ABC transporter ATPase component [*Lactobacillus johnsonii* NCC 533]	Leeber et al., 2008
Mechanisms of active response to stress related to GIT passage	AAS09244.1	hypothetical protein LJ_1476 [*Lactobacillus johnsonii* NCC 533]	Leeber et al., 2008
Mechanisms of active response to stress related to GIT passage	AAV42845.1	amino acid permease [*Lactobacillus acidophilus*]	Leeber et al., 2008
Mechanisms of active response to stress related to GIT passage	AAV43253.1	hypothetical protein LBA1428 [*Lactobacillus acidophilus* NCFM]	Leeber et al., 2008
Mechanisms of active response to stress related to GIT passage	AAV43254.1	putative transporter-membrane protein [*Lactobacillus acidophilus* NCFM]	Leeber et al., 2008
Mechanisms of active response to stress related to GIT passage	AAV43256.1	two-component response regulator [*Lactobacillus acidophilus* NCFM]	Leeber et al., 2008
Mechanisms of active response to stress related to GIT passage	AAV43257.1	hypothetical protein LBA1432 [*Lactobacillus acidophilus* NCFM]	Leeber et al., 2008

Mechanisms of active response to stress related to GIT passage	AAV43343.1	two-component sensor histidine kinase [*Lactobacillus acidophilus* NCFM]	Leeber et al., 2008
Mechanisms of active response to stress related to GIT passage	AAV43545.1	phospho-glucosyltransferase [*Lactobacillus acidophilus* NCFM]	Leeber et al., 2008
Mechanisms of active response to stress related to GIT passage	AAV43548.1	exopolysaccharide biosynthesis protein [*Lactobacillus acidophilus* NCFM]	Leeber et al., 2008
Mechanisms of active response to stress related to GIT passage	AAY41912.1	SlpA [*Lactobacillus crispatus*]	Leeber et al., 2008
Mechanisms of active response to stress related to GIT passage	ABB59702.1	EpsE [*Lactobacillus helveticus*]	Leeber et al., 2008
Mechanisms of active response to stress related to GIT passage	ABC59818.1	LuxS [*Lacticaseibacillus rhamnosus* GG]	Leeber et al., 2008
Mechanisms of active response to stress related to GIT passage	ABD96538.1	epsJ [*Streptococcus thermophilus*]	Leeber et al., 2008
Mechanisms of active response to stress related to GIT passage	ABO43774.1	ClpE [*Limosilactobacillus reuteri*]	Leeber et al., 2008
Mechanisms of active response to stress related to GIT passage	ABQ95536.1	LuxS [*Limosilactobacillus reuteri*]	Leeber et al., 2008
Mechanisms of active response to stress related to GIT passage	ABS84230.1	multidrug resistance (MDR) ABC transporter ATP-binding and permease protein, partial [*Limosilactobacillus reuteri*]	Leeber et al., 2008
Mechanisms of active response to stress related to GIT passage	ABX27751.1	LuxS [*Lactobacillus helveticus* DPC 4571]	Leeber et al., 2008
Mechanisms of active response to stress related to GIT passage	ACB28477.1	LuxS [*Streptococcus suis*]	Leeber et al., 2008
Mechanisms of active response to stress related to GIT passage	AEA56341.1	S-ribosylhomocysteinase [*Lacticaseibacillus paracasei*]	Leeber et al., 2008
Mechanisms of active response to stress related to GIT passage	AGK94888.1	Tyrosine-protein kinase EpsD [*Lactobacillus acidophilus* La-14]	Leeber et al., 2008
Mechanisms of active response to stress related to GIT passage	AGT21521.1	S-ribosylhomocysteinase luxS [*Lactiplantibacillus plantarum*]	Leeber et al., 2008
Mechanisms of active response to stress related to GIT passage	AHI39593.1	ATP-dependent Clp protease, ATP-binding subunit ClpL [*Enterococcus faecalis* DENG1]	Leeber et al., 2008

Mechanisms of active response to stress related to GIT passage	AHN70215.1	DNA starvation/stationary phase protection protein Dps [*Lactiplantibacillus plantarum* DOMLa]	Leeber et al., 2008
Mechanisms of active response to stress related to GIT passage	AJP46403.1	ornithine decarboxylase [*Lactobacillus acidophilus*]	Leeber et al., 2008
Mechanisms of active response to stress related to GIT passage	AJP46761.1	histidine kinase [*Lactobacillus acidophilus*]	Leeber et al., 2008
Mechanisms of active response to stress related to GIT passage	AJP47033.1	glycosyltransferase [*Lactobacillus acidophilus*]	Leeber et al., 2008
Mechanisms of active response to stress related to GIT passage	AKH34865.1	ClpE [*Streptococcus thermophilus*]	Leeber et al., 2008
Mechanisms of active response to stress related to GIT passage	AKM51758.1	epsH [*Limosilactobacillus fermentum* 3872]	Leeber et al., 2008
Mechanisms of active response to stress related to GIT passage	AOR73929.1	DNA starvation/stationary phase protection protein Dps [*Limosilactobacillus fermentum*]	Leeber et al., 2008
Mechanisms of active response to stress related to GIT passage	ARB50240.1	bile salt hydrolase [*Lactobacillus gasseri*]	Leeber et al., 2008
Mechanisms of active response to stress related to GIT passage	AUO27119.1	low molecular weight protein-tyrosine-phosphatase Wzb [*Limosilactobacillus fermentum*]	Leeber et al., 2008
Mechanisms of active response to stress related to GIT passage	AWD61419.1	Clp ATPase [*Limosilactobacillus reuteri*]	Leeber et al., 2008
Mechanisms of active response to stress related to GIT passage	AYN50070.1	Putative glycosyltransferase EpsE [*Lactobacillus johnsonii*]	Leeber et al., 2008
Mechanisms of active response to stress related to GIT passage	BBA82415.1	copper-transporting ATPase [*Lactiplantibacillus plantarum*]	Leeber et al., 2008
Mechanisms of active response to stress related to GIT passage	CAI97829.1	Putative fibronectin-binding protein [*Lactobacillus delbrueckii subsp. bulgaricus*]	Leeber et al., 2008
Mechanisms of active response to stress related to GIT passage	CAQ67279.1	ClpL [*Lacticaseibacillus paracasei*]	Leeber et al., 2008
Mechanisms of active response to stress related to GIT passage	CAR88394.1	ClpL [*Lacticaseibacillus paracasei*]	Leeber et al., 2008
Mechanisms of active response to stress related to GIT passage	CAX67044.1	epsA [*Lactobacillus johnsonii* FI9785]	Leeber et al., 2008

Mechanisms of active response to stress related to GIT passage	CCE46032.1	mannose specific adhesin, partial [*Limosilactobacillus fermentum*]	Leeber et al., 2008
Mechanisms of active response to stress related to GIT passage	CDF66781.1	Copper-transporting ATPase [*Lactobacillus acidophilus* DSM 20079 = JCM 1132 = NBRC 13951 = CIP 76.13]	Leeber et al., 2008
Mechanisms of active response to stress related to GIT passage	CDF67676.1	Bile salt hydrolase [*Lactobacillus acidophilus* DSM 20079 = JCM 1132 = NBRC 13951 = CIP 76.13]	Leeber et al., 2008
Mechanisms of active response to stress related to GIT passage	CDI59344.1	Bile salt hydrolase [*Lactobacillus helveticus* CIRM-BIA 951]	Leeber et al., 2008
Mechanisms of active response to stress related to GIT passage	CDR84439.1	DNA starvation/stationary phase protection protein Dps [*Lactobacillus delbrueckii subsp. lactis*]	Leeber et al., 2008
Mechanisms of active response to stress related to GIT passage	CUR37483.1	Tyrosine-protein kinase EpsD [*Limosilactobacillus reuteri*]	Leeber et al., 2008
Mechanisms of active response to stress related to GIT passage	CYV39199.1	putative ATP-dependent protease ATP-binding subunit ClpL [*Streptococcus suis*]	Leeber et al., 2008
Mechanisms of active response to stress related to GIT passage	EEU19018.1	ATP-dependent Clp protease ATP-binding subunit ClpE [*Lactobacillus crispatus* 125-2-CHN]	Leeber et al., 2008
Mechanisms of active response to stress related to GIT passage	EEW52510.1	fibronectin-binding protein (FnBP) A domain protein [*Lactobacillus iners* DSM 13335]	Leeber et al., 2008
Mechanisms of active response to stress related to GIT passage	EEW69041.1	fibronectin-binding protein (FnBP) A domain protein [*Lactobacillus helveticus* DSM 20075 = CGMCC 1.1877]	Leeber et al., 2008
Mechanisms of active response to stress related to GIT passage	EFQ44947.1	fibronectin-binding protein (FnBP) A domain protein [*Lactobacillus crispatus* CTV-05]	Leeber et al., 2008
Mechanisms of active response to stress related to GIT passage	EFQ46198.1	fibronectin-binding protein (FnBP) A domain protein [*Lactobacillus gasseri* MV-22]	Leeber et al., 2008
Mechanisms of active response to stress related to GIT passage	EGD27294.1	DNA starvation/stationary phase protection protein Dps [*Lactobacillus delbrueckii subsp. lactis* DSM 20072]	Leeber et al., 2008
Mechanisms of active response to stress related to GIT passage	EGF35484.1	copper-transporting atpase [*Lactobacillus helveticus* MTCC 5463]	Leeber et al., 2008
Mechanisms of active response to stress related to GIT passage	EHM33659.1	ClpE [*Enterococcus faecium* E4453]	Leeber et al., 2008

Mechanisms of active response to stress related to GIT passage	EHS83684.1	ATP-dependent Clp protease, ATP-binding subunit ClpE [*Lactiplantibacillus plantarum subsp. plantarum* NC8]	Leeber et al., 2008
Mechanisms of active response to stress related to GIT passage	ELA79786.1	ATP-dependent Clp protease ATP-binding subunit ClpL [*Enterococcus faecium* EnGen0004]	Leeber et al., 2008
Mechanisms of active response to stress related to GIT passage	ERL65647.1	ClpC [*Schleiferilactobacillus shenzhenensis* LY-73]	Leeber et al., 2008
Mechanisms of active response to stress related to GIT passage	ERL66139.1	ClpE [*Schleiferilactobacillus shenzhenensis* LY-73]	Leeber et al., 2008
Mechanisms of active response to stress related to GIT passage	GAN41145.1	copper-transporting ATPase [*Lacticaseibacillus paracasei* NRIC 1981]	Leeber et al., 2008
Mechanisms of active response to stress related to GIT passage	GDZ84074.1	S-ribosylhomocysteine lyase luxS [*Leuconostoc citreum*]	Leeber et al., 2008
Mechanisms of active response to stress related to GIT passage	GEL33985.1	ATP-dependent Clp protease ATP-binding subunit ClpC [*Lactiplantibacillus plantarum subsp. plantarum*]	Leeber et al., 2008
Mechanisms of active response to stress related to GIT passage	GFP01202.1	cell division protein [*Lactobacillus helveticus*]	Leeber et al., 2008
Mechanisms of active response to stress related to GIT passage	GFP15270.1	ATP-dependent Clp protease ATP-binding subunit ClpC [*Lactobacillus helveticus*]	Leeber et al., 2008
Mechanisms of active response to stress related to GIT passage	GHN19320.1	ATP-dependent Clp protease ATP-binding subunit [*Lactobacillus delbrueckii*]	Leeber et al., 2008
Mechanisms of active response to stress related to GIT passage	KPH21867.1	copper-transporting ATPase [*Limosilactobacillus fermentum*]	Leeber et al., 2008
Mechanisms of active response to stress related to GIT passage	KRK29711.1	mucus binding protein [*Lactobacillus acidophilus* DSM 20079 = JCM 1132 = NBRC 13951 = CIP 76.13]	Leeber et al., 2008
Mechanisms of active response to stress related to GIT passage	KRK44693.1	copper-transporting ATPase [*Lactobacillus amylovorus* DSM 20531]	Leeber et al., 2008
Mechanisms of active response to stress related to GIT passage	KRK59947.1	DNA starvation stationary phase protection protein Dps [*Limosilactobacillus antri* DSM 16041]	Leeber et al., 2008
Mechanisms of active response to stress related to GIT passage	KRK74393.1	DNA starvation stationary phase protection protein Dps [*Lacticaseibacillus nasuensis* JCM 17158]	Leeber et al., 2008

Mechanisms of active response to stress related to GIT passage	KRM84632.1	DNA starvation stationary phase protection protein Dps [*Lactobacillus hominis* DSM 23910 = CRBIP 24.179]	Leeber et al., 2008
Mechanisms of active response to stress related to GIT passage	PMC47034.1	DNA starvation/stationary phase protection protein Dps [*Lactobacillus iners*]	Leeber et al., 2008
Mechanisms of active response to stress related to GIT passage	QAS50172.1	ATP-dependent Clp protease ATP-binding subunit ClpC [*Latilactobacillus curvatus* JCM 1096 = DSM 20019]	Leeber et al., 2008
Mechanisms of active response to stress related to GIT passage	QHM38919.1	putative sugar transferase EpsL (plasmid) [*Lactiplantibacillus plantarum*]	Leeber et al., 2008
Mechanisms of active response to stress related to GIT passage	QPP16258.1	glutamate:gamma-aminobutyrate (Glu/GABA) antiporter [*Lactobacillus crispatus*]	Leeber et al., 2008
Mechanisms of active response to stress related to GIT passage	RNE38687.1	putative sugar transferase EpsL [*Lacticaseibacillus paracasei*]	Leeber et al., 2008
Mechanisms of active response to stress related to GIT passage	SPS15181.1	putative sugar transferase EpsL [*Lactobacillus helveticus*]	Leeber et al., 2008
Mechanisms of active response to stress related to GIT passage	SPX68388.1	ATP-dependent Clp protease ATP-binding subunit ClpL [*Lactiplantibacillus plantarum subsp. plantarum*]	Leeber et al., 2008
Mechanisms of active response to stress related to GIT passage	UHX60220.1	bile salt hydrolase [*Lactobacillus johnsonii*]	Leeber et al., 2008
Mechanisms of active response to stress related to GIT passage	UUZ04888.1	BSHa [*Lactobacillus johnsonii*]	Leeber et al., 2008
Mechanisms of active response to stress related to GIT passage	UZZ85054.1	S-layer protein [*Lactobacillus crispatus*]	Leeber et al., 2008
Mechanisms of active response to stress related to GIT passage	WP_019252340.1	D-alanyl-lipoteichoic acid biosynthesis protein DltD [*Limosilactobacillus reuteri*]	Leeber et al., 2008
Mechanisms of active response to stress related to GIT passage	WP_019898995.1	peptide-methionine (R)-S-oxide reductase MsrB [*Lacticaseibacillus paracasei*]	Leeber et al., 2008
Mechanisms of active response to stress related to GIT passage	WP_048588304.1	D-alanine--poly(phosphoribitol) ligase subunit DltA [*Lactobacillus*]	Leeber et al., 2008
Mechanisms of active response to stress related to GIT passage	WP_104142777.1	peptide-methionine (R)-S-oxide reductase MsrB [*Lactococcus lactis*]	Leeber et al., 2008
Mechanisms of active response to stress related to GIT passage	WP_104832728.1	peptide-methionine (R)-S-oxide reductase MsrB [*Enterococcus faecium*]	Leeber et al., 2008

Mechanisms of active response to stress related to GIT passage	WP_109241568.1	peptide-methionine (R)-S-oxide reductase MsrB [*Ligilactobacillus salivarius*]	Leeber et al., 2008
Mechanisms of active response to stress related to GIT passage	WP_109918411.1	peptide-methionine (R)-S-oxide reductase MsrB [*Lactobacillus*]	Leeber et al., 2008
Mechanisms of active response to stress related to GIT passage	WP_118980074.1	peptide-methionine (R)-S-oxide reductase MsrB [*Lactobacillus delbrueckii*]	Leeber et al., 2008
Mechanisms of active response to stress related to GIT passage	WP_153232390.1	peptide-methionine (R)-S-oxide reductase MsrB [*Lactobacillus gasseri*]	Leeber et al., 2008
Mechanisms of active response to stress related to GIT passage	WP_195212129.1	adhesin [*Lactobacillus*]	Leeber et al., 2008
Mechanisms of active response to stress related to GIT passage	WP_220437225.1	peptide-methionine (R)-S-oxide reductase MsrB [*Lactococcus raffinolactis*]	Leeber et al., 2008
Mechanisms of active response to stress related to GIT passage	WP_229036516.1	adhesin [*Lactobacillus*]	Leeber et al., 2008
Shikimate and folate biosynthesis pathway (CHORISMATE)	QJU49979.1	3-deoxy-7-phosphoheptulonate synthase [*Lactiplantibacillus paraplantarum*]	D'Aimmo et al., 2023
Shikimate and folate biosynthesis pathway (CHORISMATE)	SFE47508.1	3-dehydroquinate synthase [*Lactobacillus rogosae*]	D'Aimmo et al., 2023
Shikimate and folate biosynthesis pathway (CHORISMATE)	EIW13109.1	3-dehydroquinate dehydratase I [*Lactiplantibacillus pentosus* KCA1]	D'Aimmo et al., 2023
Shikimate and folate biosynthesis pathway (CHORISMATE)	WP_172985744.1	Shikimate dehydrogenase [*Lactobacillus*]	D'Aimmo et al., 2023
Shikimate and folate biosynthesis pathway (CHORISMATE)	WP_072544160.1	Shikimate kinase [*Lactobacillus*]	D'Aimmo et al., 2023
Shikimate and folate biosynthesis pathway (CHORISMATE)	CUS17452.1	3-phosphoshikimate 1-carboxyvinyltransferase [*Lactobacillus delbrueckii subsp. bulgaricus*]	D'Aimmo et al., 2023
Shikimate and folate biosynthesis pathway (CHORISMATE)	WP_231128327.1	Chorismate synthase [*Lactobacillus*]	D'Aimmo et al., 2023

Shikimate and folate biosynthesis pathway (para-aminobenzoic acid, PABA)	QJU49551.1	Aminodeoxychorismate synthase [*Lactiplantibacillus paraplantarum*]	D'Aimmo et al., 2023
Shikimate and folate biosynthesis pathway (para-aminobenzoic acid, PABA)	GBA87487.1	Aminodeoxychorismate lyase [*Lactobacillus paragasseri*]	D'Aimmo et al., 2023
Shikimate and folate biosynthesis pathway (6-hydroxymethyl-7,8-dihydropterin pyrophosphate, DHPPP)	QAA29097.1	Dihydroneopterin triphosphate diphosphatase [*Lactiplantibacillus plantarum*]	D'Aimmo et al., 2023
Shikimate and folate biosynthesis pathway (6-hydroxymethyl-7,8-dihydropterin pyrophosphate, DHPPP)	WP_261937608.1	Dihydroneopterin aldolase [*Lactobacillus*]	D'Aimmo et al., 2023
Shikimate and folate biosynthesis pathway (6-hydroxymethyl-7,8-dihydropterin pyrophosphate, DHPPP)	KRL59967.1	2-amino-4-hydroxy-6-hydroxymethyldihydropteridine diphosphokinase [*Lactobacillus*]	D'Aimmo et al., 2023
Shikimate and folate biosynthesis pathway (tetrahydrofolate, THF)	WP_172985600.1	Dihydropteroate synthase [*Lactobacillus*]	D'Aimmo et al., 2023
Shikimate and folate biosynthesis pathway (tetrahydrofolate, THF)	QJU51066.1	Dihydrofolate synthase [*Lactiplantibacillus paraplantarum*]	D'Aimmo et al., 2023
Shikimate and folate biosynthesis pathway (tetrahydrofolate, THF)	KRK54541.1	Tetrahydrofolate synthase [*Lactobacillus johnsonii* ATCC 33200]	D'Aimmo et al., 2023
Shikimate and folate biosynthesis pathway (tetrahydrofolate, THF)	WP_172985207.1	Dihydrofolate reductase [*Lactobacillus*]	D'Aimmo et al., 2023
Tryptophan pathway	ASW11534.1	Aromatic amino acid aminotransferase [*Lactobacillus delbrueckii subsp. lactis*]	Pan et al., 2023
Tryptophan pathway	ALT48283.1	Lactate dehydrogenase [*Lactobacillus delbrueckii subsp. lactis*]	Pan et al., 2023
Tryptophan pathway	sp|J7SHB8.1|	Indolelactate dehydrogenase [*Clostridium sporogenes*]	Pan et al., 2023
Tryptophan pathway	VDG20848.1	Cinnamoyl-coa--phenyllactate coa-transferase [*Lactiplantibacillus mudanjiangensis*]	Pan et al., 2023
Tryptophan pathway	sp|Q93AM0.1|	Phenyllactate dehydratase activator [*Clostridium sporogenes*]	Pan et al., 2023

Tryptophan pathway	GEK41904.1	Indolepyruvate decarboxylase [*Ligilactobacillus aviarius*]	Pan et al., 2023
Tryptophan pathway	AEO49255.1	Phenylpyruvate decarboxylase [*Rhodospirillum rubrum*]	Pan et al., 2023
Tryptophan pathway	GHN33536.1	Aldehyde dehydrogenase [*Lactobacillus delbrueckii*]	Pan et al., 2023
Tryptophan pathway	POD81577.1	Tryptophan 2-monooxygenase [*Lactiplantibacillus paraplantarum*]	Pan et al., 2023
Tryptophan pathway	GEB91345.1	Amidase [*Lactobacillus delbrueckii subsp. lactis*]	Pan et al., 2023
Tryptophan pathway	WPX87954.1	Tryptophan decarboxylase [*Pseudomonas asiatica*]	Pan et al., 2023
Tryptophan pathway	BEC98836.1	Diamine oxidase [*Escherichia coli*]	Pan et al., 2023
Tryptophan pathway	sp|J7TF92.1|	3-(aryl)acrylate reductase [*Clostridium sporogenes*]	Pan et al., 2023
Tryptophan pathway	KAA9236053.1	Copper amine oxidase [*Lactobacillus jensenii*]	Pan et al., 2023
Host Interaction and Immunomodulation (estrogen deconjugation)	WP_102732122.1	Glycosyl hydrolase family 2 (GH2) [*A. muciniphila*]	(Ervin et al., 2019)
Host Interaction and Immunomodulation (estrogen deconjugation)	WP_102727023.1	Glycosyl hydrolase family 2 (GH2) [*Akkermansia* sp. CAG: 344]	(Ervin et al., 2019)
Host Interaction and Immunomodulation (estrogen deconjugation)	CDA71244.1	Glycosyl hydrolase family 2 (GH2) [*B. coprocola*]	(Ervin et al., 2019)
Host Interaction and Immunomodulation (estrogen deconjugation)	WP_130053801.1	Glycosyl hydrolase family 2 (GH2) [*B. dorei*]	(Ervin et al., 2019)
Host Interaction and Immunomodulation (estrogen deconjugation)	WP_005822521.1	Glycosyl hydrolase family 2 (GH2) [*B. fragilis*]	(Ervin et al., 2019)
Host Interaction and Immunomodulation (estrogen deconjugation)	WP_055167711.1	Glycosyl hydrolase family 2 (GH2) [*Bacteroides spp.*]	(Ervin et al., 2019)
Host Interaction and Immunomodulation (estrogen deconjugation)	WP_025866948.1	Glycosyl hydrolase family 2 (GH2) [*B. nordii*]	(Ervin et al., 2019)
Host Interaction and Immunomodulation (estrogen deconjugation)	EDO11197.1	Glycosyl hydrolase family 2 (GH2) [*B. ovatus*]	(Ervin et al., 2019)

Host Interaction and Immunomodulation (estrogen deconjugation)	WP_004298526.1	Glycosyl hydrolase family 2 (GH2) [*B. ovatus*]	(Ervin et al., 2019)
Host Interaction and Immunomodulation (estrogen deconjugation)	WP_117588613.1	Glycosyl hydrolase family 2 (GH2) [*Bacteroides*]	(Ervin et al., 2019)
Host Interaction and Immunomodulation (estrogen deconjugation)	WP_122375735.1	Glycosyl hydrolase family 2 (GH2) [*Parabacteroides*]	(Ervin et al., 2019)
Host Interaction and Immunomodulation (estrogen deconjugation)	WP_016272112.1	Glycosyl hydrolase family 2 (GH2) [*Bacteroides*]	(Ervin et al., 2019)
Host Interaction and Immunomodulation (estrogen deconjugation)	WP_022502167.1	beta-glucuronidase (GUS) [*Lachnospira*]	(Ervin et al., 2019)
Host Interaction and Immunomodulation (estrogen deconjugation)	WP_066714276.1	beta-glucuronidase (GUS) [*Clostridium* sp. Marseille-P299]	(Ervin et al., 2019)
Host Interaction and Immunomodulation (estrogen deconjugation)	WP_001551153.1	beta-glucuronidase (GUS) [*Enterobacteriaceae*]	(Ervin et al., 2019)
Host Interaction and Immunomodulation (estrogen deconjugation)	CDA40053.1	Glycosyl hydrolase family 2 (GH2) [*Eubacterium eligens*]	(Ervin et al., 2019)
Host Interaction and Immunomodulation (estrogen deconjugation)	HCG85850.1	Glycosyl hydrolase family 2 (GH2) [*Faecalibacterium*]	(Ervin et al., 2019)
Host Interaction and Immunomodulation (estrogen deconjugation)	SCH31850.1	beta-glucuronidase (GUS) [uncultured *Faecalibacterium sp.*]	(Ervin et al., 2019)
Host Interaction and Immunomodulation (estrogen deconjugation)	WP_097774993. 1	beta-glucuronidase (GUS) [*F. prausnitzii*]	(Ervin et al., 2019)
Host Interaction and Immunomodulation (estrogen deconjugation)	WP_069152983.1	Glycosyl hydrolase family 2 (GH2) [*Eisenbergiella tayi*]	(Ervin et al., 2019)
Host Interaction and Immunomodulation (estrogen deconjugation)	CBK98066.1	beta-glucuronidase (GUS) [*F. prausnitzii*]	(Ervin et al., 2019)
Host Interaction and Immunomodulation (estrogen deconjugation)	CBJ55484.1	beta-glucuronidase (GUS) [uncultured bacterium]	(Ervin et al., 2019)
Host Interaction and Immunomodulation (estrogen deconjugation)	HAN02355.1	beta-glucuronidase (GUS) [*Lactobacillus sp.]*	(Ervin et al., 2019)
Host Interaction and Immunomodulation (estrogen deconjugation)	HBH98519.1	beta-glucuronidase (GUS) [*L. rhamnosus*]	(Ervin et al., 2019)
Host Interaction and Immunomodulation (estrogen deconjugation)	WP_122205889.1	Glycosyl hydrolase family 2 (GH2) [*Parabacteroides*]	(Ervin et al., 2019)

Host Interaction and Immunomodulation (estrogen deconjugation)	WP_010800018.1	Glycosyl hydrolase family 2 (GH2) [*Prevotella*]	(Ervin et al., 2019)
Host Interaction and Immunomodulation (estrogen deconjugation)	WP_118445354.1	beta-glucuronidase (GUS) [*Ruminococcus gnavus*]	(Ervin et al., 2019)
Host Interaction and Immunomodulation (estrogen deconjugation)	WP_118587093	Glycosyl hydrolase family 2 (GH2) [*Roseburia inulinivorans*]	(Ervin et al., 2019)
Host Interaction and Immunomodulation (estrogen deconjugation)	WP_118581144.1	Glycosyl hydrolase family 2 (GH2) [*Roseburia inulinivorans*]	(Ervin et al., 2019)
Host Interaction and Immunomodulation (estrogen deconjugation)	WP_118096903.1	Glycosyl hydrolase family 2 (GH2) [*R. hominis*]	(Ervin et al., 2019)
Host Interaction and Immunomodulation (estrogen deconjugation)	WP_055167711.1	Glycosyl hydrolase family 2 (GH2) [*Clostridia*]	(Ervin et al., 2019)
Host Interaction and Immunomodulation (estrogen deconjugation)	CDE36900.1	Glycosyl hydrolase family 2 (GH2) [*Eubacterium*]	(Ervin et al., 2019)
Host Interaction and Immunomodulation (estrogen deconjugation)	CDE18721.1	beta-glucuronidase (GUS) [*Acidiphilium sp.*]	(Ervin et al., 2019)
Host Interaction and Immunomodulation (Steroid hormones degradation)	pdb|1HXH|A	Chain A, 3BETA/17BETA-HYDROXYSTEROID DEHYDROGENASE [*Comamonas testosteroni*]	(Li et al., 2021)
Host Interaction and Immunomodulation (Steroid hormones degradation)	pdb|1HXH|B	Chain B, 3BETA/17BETA-HYDROXYSTEROID DEHYDROGENASE [*Comamonas testosteroni*]	(Li et al., 2021)
Host Interaction and Immunomodulation (Steroid hormones degradation)	pdb|1HXH|C	Chain C, 3BETA/17BETA-HYDROXYSTEROID DEHYDROGENASE [*Comamonas testosteroni*]	(Li et al., 2021)
Host Interaction and Immunomodulation (Steroid hormones degradation)	pdb|1HXH|D	Chain D, 3BETA/17BETA-HYDROXYSTEROID DEHYDROGENASE [*Comamonas testosteroni*]	(Li et al., 2021)
