# Supplementary material for: Women’s health is a team effort: probiogenomics supports the development of a multi-species vaginal probiotic
Source: Cell Mol Life Sci. 2026 Feb 26;83(1):132. doi: 10.1007/s00018-026-06107-2 (PMC12957687; doi:10.1007/s00018-026-06107-2)

Women's health is a team effort: probiogenomics supports the development of a multi-species vaginal probiotic

Chiara Maria Calvanese[1], Vincenzo Valentino[1], Annachiara De Prisco[2], Serena Allesina[2], Angela Amoruso[2], Francesca Deidda[2], Annalisa Visciglia[2], Danilo Ercolini[1,3], Marco Pane[2], Francesca De Filippis[1,3*]

[1] Department of Agricultural Sciences, University of Naples Federico II, P.zza Carlo di Borbone 1, 80055 Portici (NA), Italy

[2]Probiotical Research S.r.l., via Enrico Mattei 3, 28100 Novara, Italy

[3] Task Force on Microbiome Studies, University of Naples Federico II, Corso Umberto I 43, 80100 Napoli, Italy

Journal: Cellular and Molecular Life Sciences

Corresponding Author:

Prof. Francesca De Filippis

Department of Agricultural Sciences, University of Naples Federico II, Via Università 100, 80055 Portici, Italy

e-mail: francesca.defilippis@unina.it; Phone: +39 081-2539388

ORCID: 0000-0002-3474-2884

Online Resource 3. Venn diagram summarizing the number of core-soft core genes (a) and shell/cloud genes (b) in vaginal genomes, and the GO terms involved. Most important GOs associated with defense from pathogens are highlighted.

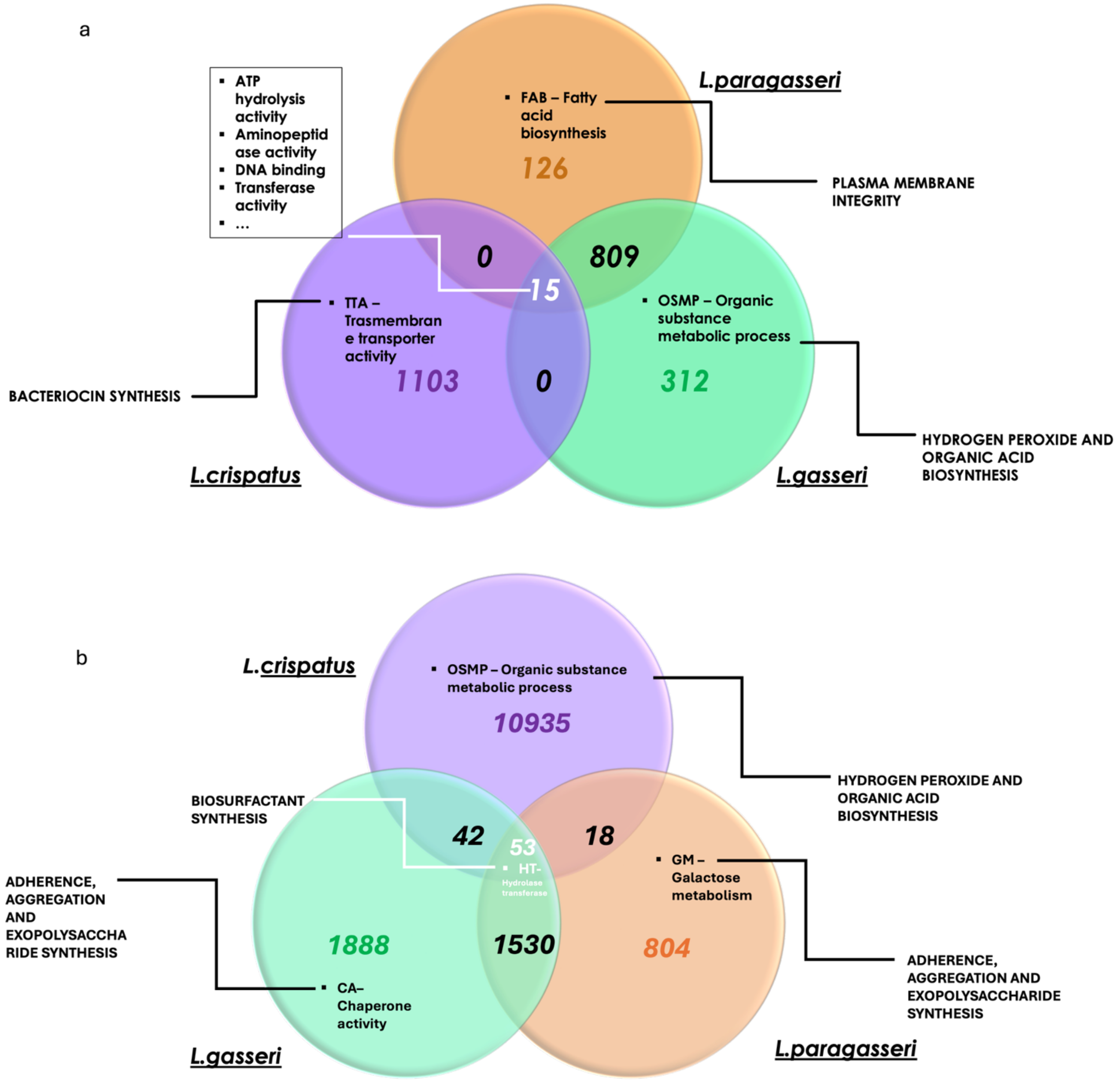

Supplement: Supplementary file 3 — Supplementary Material 3 (PDF 628 KB) [file 18_2026_6107_MOESM3_ESM.pdf]
