# Supplementary material for: Women’s health is a team effort: probiogenomics supports the development of a multi-species vaginal probiotic
Source: Cell Mol Life Sci. 2026 Feb 26;83(1):132. doi: 10.1007/s00018-026-06107-2 (PMC12957687; doi:10.1007/s00018-026-06107-2)
Supplement: Supplementary file 4 — Supplementary Material 4 (PDF 159 KB) [file 18_2026_6107_MOESM4_ESM.pdf]

Women's health is a team effort: probiogenomics supports the development of a multi-species vaginal probiotic

Chiara Maria Calvanese[1], Vincenzo Valentino[1], Annachiara De Prisco[2], Serena Allesina[2], Angela Amoruso[2], Francesca Deidda[2], Annalisa Visciglia[2], Danilo Ercolini[1,3], Marco Pane[2], Francesca De Filippis[1,3*]

[1] Department of Agricultural Sciences, University of Naples Federico II, P.zza Carlo di Borbone 1, 80055 Portici (NA), Italy

[2]Probiotical Research S.r.l., via Enrico Mattei 3, 28100 Novara, Italy

[3] Task Force on Microbiome Studies, University of Naples Federico II, Corso Umberto I 43, 80100 Napoli, Italy

Journal: Cellular and Molecular Life Sciences

Corresponding Author:

Prof. Francesca De Filippis

Department of Agricultural Sciences, University of Naples Federico II, Via Università 100, 80055 Portici, Italy

e-mail: francesca.defilippis@unina.it; Phone: +39 081-2539388

ORCID: 0000-0002-3474-2884

Online Resource 4. Key GOs found in the vaginal pangenomes

Key GOs found in core genes of vaginal genomes

**Species**	**GO terms**	**Molecular function/ Biological process**	**Key function**
L. paragasseri	GO:0006012	GM - Galactose metabolism	Adherence, aggregation, and exopolysaccharide synthesis
L. paragasseri	GO:0022857	PTTA – passive transmembrane transporter activity	Adherence, aggregation, and exopolysaccharide synthesis
L. paragasseri	GO:0097367	CDB – Carbohydrate derivative binding	Adherence, aggregation, and exopolysaccharide synthesis
L. paragasseri	GO:0006457	CA - Chaperone activity	Adherence, aggregation, and exopolysaccharide synthesis
L. paragasseri	GO:0140359	ABC transporter	Bacteriocin synthesis
L. paragasseri	GO:0008324	TTA – Transmembrane transporter activity	Bacteriocin synthesis
L. paragasseri	GO:1901135	GCMP – Glycosyl compound metabolic process	Biosurfactant synthesis
L. paragasseri	GO:0016787	Hydrolase transferase	Biosurfactant synthesis
L. paragasseri	GO:0006631	FAB – Fatty acid biosynthesis	Plasma membrane integrity
L. paragasseri	GO:0006633	FAB – Fatty acid biosynthesis	Plasma membrane integrity
L. gasseri	GO:0006012	GM - Galactose metabolism	Adherence, aggregation, and exopolysaccharide synthesis
L. gasseri	GO:0006457	CA - Chaperone activity	Adherence, aggregation, and exopolysaccharide synthesis
L. gasseri	GO:0022857	PTTA – passive transmembrane transporter activity	Adherence, aggregation, and exopolysaccharide synthesis
L. gasseri	GO:0097367	CDB – Carbohydrate derivative binding	Adherence, aggregation, and exopolysaccharide synthesis
L. gasseri	GO:0008324	TTA – Transmembrane transporter activity	Bacteriocin synthesis
L. gasseri	GO:0140359	ABC transporter	Bacteriocin synthesis
L. gasseri	GO:0016787	Hydrolase transferase	Biosurfactant synthesis
L. gasseri	GO:1901135	GCMP – Glycosyl compound metabolic process	Biosurfactant synthesis
L. gasseri	GO:0008152	OSMP – Organic substance metabolic process	Hydrogen peroxide and organic acid biosynthesis<br>D/L-lactate metabolic processes
L. gasseri	GO:0006631	FAB – Fatty acid biosynthesis	Plasma membrane integrity
L. gasseri	GO:0006633	FAB – Fatty acid biosynthesis	Plasma membrane integrity

L. gasseri/L.paragasseri	GO:0006012	GM - Galactose metabolism	Adherence, aggregation, and exopolysaccharide synthesis
L. gasseri/L.paragasseri	GO:0006457	CA - Chaperone activity	Adherence, aggregation, and exopolysaccharide synthesis
L. gasseri/L.paragasseri	GO:0022857	PTTA – passive transmembrane transporter activity	Adherence, aggregation, and exopolysaccharide synthesis
L. gasseri/L.paragasseri	GO:0097367	CDB – Carbohydrate derivative binding	Adherence, aggregation, and exopolysaccharide synthesis
L. gasseri/L.paragasseri	GO:0008324	TTA – Transmembrane transporter activity	Bacteriocin synthesis
L. gasseri/L.paragasseri	GO:0140359	ABC transporter	Bacteriocin synthesis
L. gasseri/L.paragasseri	GO:0016787	Hydrolase transferase	Biosurfactant synthesis
L. gasseri/L.paragasseri	GO:1901135	GCMP – Glycosyl compound metabolic process	Biosurfactant synthesis
L. gasseri/L.paragasseri	GO:0008152	OSMP – Organic substance metabolic process	Hydrogen peroxide and organic acid biosynthesis<br>D/L-lactate metabolic processes
L. gasseri/L.paragasseri	GO:0006631	FAB – Fatty acid biosynthesis	Plasma membrane integrity
L. gasseri/L.paragasseri	GO:0006633	FAB – Fatty acid biosynthesis	Plasma membrane integrity
L.crispatus	GO:0006012	GM - Galactose metabolism	Adherence, aggregation, and exopolysaccharide synthesis
L.crispatus	GO:0006457	CA - Chaperone activity	Adherence, aggregation, and exopolysaccharide synthesis
L.crispatus	GO:0022857	PTTA – passive transmembrane transporter activity	Adherence, aggregation, and exopolysaccharide synthesis
L.crispatus	GO:0097367	CDB – Carbohydrate derivative binding	Adherence, aggregation, and exopolysaccharide synthesis
L.crispatus	GO:0008324	TTA – Transmembrane transporter activity	Bacteriocin synthesis
L.crispatus	GO:0140359	ABC transporter	Bacteriocin synthesis
L.crispatus	GO:0016787	Hydrolase transferase	Biosurfactant synthesis
L.crispatus	GO:1901135	GCMP – Glycosyl compound metabolic process	Biosurfactant synthesis

L.crispatus	GO:0008152	OSMP – Organic substance metabolic process	Hydrogen peroxide and organic acid biosynthesis<br>D/L-lactate metabolic processes
L.crispatus	GO:0006631	FAB – Fatty acid biosynthesis	Plasma membrane integrity
L.crispatus	GO:0006633	FAB – Fatty acid biosynthesis	Plasma membrane integrity
L. gasseri/L.paragasseri/L.crispatus	GO:0004177	Basic survival functions	aminopeptidase activity
L. gasseri/L.paragasseri/L.crispatus	GO:0004816	Basic survival functions	asparagine-tRNA ligase activity
L. gasseri/L.paragasseri/L.crispatus	GO:0006421	Basic survival functions	asparaginyl-tRNA aminoacylation
L. gasseri/L.paragasseri/L.crispatus	GO:0005524	Basic survival functions	ATP binding
L. gasseri/L.paragasseri/L.crispatus	GO:0005524	Basic survival functions	ATP binding
L. gasseri/L.paragasseri/L.crispatus	GO:0005524	Basic survival functions	ATP binding
L. gasseri/L.paragasseri/L.crispatus	GO:0016887	Basic survival functions	ATP hydrolysis activity
L. gasseri/L.paragasseri/L.crispatus	GO:0004176	Basic survival functions	ATP-dependent peptidase
L. gasseri/L.paragasseri/L.crispatus	GO:0009986	Basic survival functions	cell surface
L. gasseri/L.paragasseri/L.crispatus	GO:0005737	Basic survival functions	cytoplasm
L. gasseri/L.paragasseri/L.crispatus	GO:0005737	Basic survival functions	cytoplasm
L. gasseri/L.paragasseri/L.crispatus	GO:0005737	Basic survival functions	cytoplasm
L. gasseri/L.paragasseri/L.crispatus	GO:0005737	Basic survival functions	cytoplasm
L. gasseri/L.paragasseri/L.crispatus	GO:0005737	Basic survival functions	cytoplasm

L. gasseri/L.paragasseri/L.crispatus	GO:0003677	Basic survival functions	DNA binding
L. gasseri/L.paragasseri/L.crispatus	GO:0031975	Basic survival functions	envelope
L. gasseri/L.paragasseri/L.crispatus	GO:0005576	Basic survival functions	extracellular region
L. gasseri/L.paragasseri/L.crispatus	GO:0006096	Basic survival functions	glycolytic process
L. gasseri/L.paragasseri/L.crispatus	GO:0015934	Basic survival functions	large ribosomal subunit
L. gasseri/L.paragasseri/L.crispatus	GO:0000287	Basic survival functions	magnesium ion binding
L. gasseri/L.paragasseri/L.crispatus	GO:0008168	Basic survival functions	methyltransferase activity
L. gasseri/L.paragasseri/L.crispatus	GO:0051287	Basic survival functions	NAD binding
L. gasseri/L.paragasseri/L.crispatus	GO:0045892	Basic survival functions	negative regulation of DNA-templated transcription
L. gasseri/L.paragasseri/L.crispatus	GO:0016616	Basic survival functions	oxidoreductase activity, acting on the CH-OH group of donors, NAD or NADP as acceptor
L. gasseri/L.paragasseri/L.crispatus	GO:0004634	Basic survival functions	phosphopyruvate hydratase activity
L. gasseri/L.paragasseri/L.crispatus	GO:0006508	Basic survival functions	proteolysis
L. gasseri/L.paragasseri/L.crispatus	GO:0006508	Basic survival functions	proteolysis
L. gasseri/L.paragasseri/L.crispatus	GO:1990904	Basic survival functions	ribonucleoprotein complex
L. gasseri/L.paragasseri/L.crispatus	GO:1990904	Basic survival functions	ribonucleoprotein complex
L. gasseri/L.paragasseri/L.crispatus	GO:0043023	Basic survival functions	ribosomal large subunit binding
L. gasseri/L.paragasseri/L.crispatus	GO:0005840	Basic survival functions	ribosome

L. gasseri/L.paragasseri/L.crispatus	GO:0005840	Basic survival functions	ribosome
L. gasseri/L.paragasseri/L.crispatus	GO:0043022	Basic survival functions	ribosome binding
L. gasseri/L.paragasseri/L.crispatus	GO:0001510	Basic survival functions	RNA methylation
L. gasseri/L.paragasseri/L.crispatus	GO:0019843	Basic survival functions	rRNA binding
L. gasseri/L.paragasseri/L.crispatus	GO:0019843	Basic survival functions	rRNA binding
L. gasseri/L.paragasseri/L.crispatus	GO:0019843	Basic survival functions	rRNA binding
L. gasseri/L.paragasseri/L.crispatus	GO:0004252	Basic survival functions	serine-type endopeptidase activity
L. gasseri/L.paragasseri/L.crispatus	GO:0003735	Basic survival functions	structural constituent of ribosome
L. gasseri/L.paragasseri/L.crispatus	GO:0003735	Basic survival functions	structural constituent of ribosome
L. gasseri/L.paragasseri/L.crispatus	GO:0016740	Basic survival functions	transferase activity
L. gasseri/L.paragasseri/L.crispatus	GO:0006412	Basic survival functions	translation
L. gasseri/L.paragasseri/L.crispatus	GO:0006412	Basic survival functions	translation
L. gasseri/L.paragasseri/L.crispatus	GO:0006412	Basic survival functions	translation
L. gasseri/L.paragasseri/L.crispatus	GO:0003746	Basic survival functions	translation elongation factor activity
L. gasseri/L.paragasseri/L.crispatus	GO:0003743	Basic survival functions	translation initiation factor activity
L. gasseri/L.paragasseri/L.crispatus	GO:0008270	Basic survival functions	zinc ion binding

Key GOs found in cloud genes of vaginal genomes

Species	GO terms	Molecular function/ Biological process	Key function
L. paragasseri	GO:0006012	GM - Galactose metabolism	Adherence, aggregation, and exopolysaccharide synthesis
L. paragasseri	GO:0006457	CA - Chaperone activity	Adherence, aggregation, and exopolysaccharide synthesis
L. paragasseri	GO:0019318	GM - Galactose metabolism	Adherence, aggregation, and exopolysaccharide synthesis
L. paragasseri	GO:0022857	PTTA – passive transmembrane transporter activity	Adherence, aggregation, and exopolysaccharide synthesis
L. paragasseri	GO:0097367	CDB – Carbohydrate derivative binding	Adherence, aggregation, and exopolysaccharide synthesis
L. paragasseri	GO:0008324	TTA – Transmembrane transporter activity	Bacteriocin synthesis
L. paragasseri	GO:0140359	ABC transporter	Bacteriocin synthesis
L. paragasseri	GO:0016787	Hydrolase transferase	Biosurfactant synthesis
L. paragasseri	GO:1901135	GCMP – Glycosyl compound metabolic process	Biosurfactant synthesis
L. paragasseri	GO:0008152	OSMP – Organic substance metabolic process	Hydrogen peroxide and organic acid biosynthesis<br>D/L-lactate metabolic processes
L. gasseri	GO:0006012	GM - Galactose metabolism	Adherence, aggregation, and exopolysaccharide synthesis
L. gasseri	GO:0006457	CA - Chaperone activity	Adherence, aggregation, and exopolysaccharide synthesis
L. gasseri	GO:0022857	PTTA – passive transmembrane transporter activity	Adherence, aggregation, and exopolysaccharide synthesis
L. gasseri	GO:0097367	CDB – Carbohydrate derivative binding	Adherence, aggregation, and exopolysaccharide synthesis
L. gasseri	GO:0008324	TTA – Transmembrane transporter activity	Bacteriocin synthesis
L. gasseri	GO:0140359	ABC transporter	Bacteriocin synthesis
L. gasseri	GO:0016787	Hydrolase transferase	Biosurfactant synthesis
L. gasseri	GO:1901135	GCMP – Glycosyl compound metabolic process	Biosurfactant synthesis
L. gasseri	GO:0008152	OSMP – Organic substance metabolic process	Hydrogen peroxide and organic acid biosynthesis<br>D/L-lactate metabolic processes
L. gasseri	GO:0071704	OSMP – Organic substance metabolic process	Hydrogen peroxide and organic acid biosynthesis<br>D/L-lactate metabolic processes
L. gasseri	GO:0006631	FAB – Fatty acid biosynthesis	Plasma membrane integrity

L. gasseri	GO:0006633	FAB – Fatty acid biosynthesis	Plasma membrane integrity
L. crispatus	GO:0006012	GM - Galactose metabolism	Adherence, aggregation, and exopolysaccharide synthesis
L. crispatus	GO:0006457	CA - Chaperone activity	Adherence, aggregation, and exopolysaccharide synthesis
L. crispatus	GO:0022857	PTTA – passive transmembrane transporter activity	Adherence, aggregation, and exopolysaccharide synthesis
L. crispatus	GO:0097367	CDB – Carbohydrate derivative binding	Adherence, aggregation, and exopolysaccharide synthesis
L. crispatus	GO:0008324	TTA – Transmembrane transporter activity	Bacteriocin synthesis
L. crispatus	GO:0140359	ABC transporter	Bacteriocin synthesis
L. crispatus	GO:0016787	Hydrolase transferase	Biosurfactant synthesis
L. crispatus	GO:1901135	GCMP – Glycosyl compound metabolic process	Biosurfactant synthesis
L. crispatus	GO:0008152	OSMP – Organic substance metabolic process	Hydrogen peroxide and organic acid biosynthesis<br>D/L-lactate metabolic processes
L. crispatus	GO:0071704	OSMP – Organic substance metabolic process	Hydrogen peroxide and organic acid biosynthesis<br>D/L-lactate metabolic processes
L. crispatus	GO:0006631	FAB – Fatty acid biosynthesis	Plasma membrane integrity
L. crispatus	GO:0006633	FAB – Fatty acid biosynthesis	Plasma membrane integrity
L. crispatus/L.gasseri	GO:0016787	Hydrolase transferase	Biosurfactant synthesis
L. crispatus/L.gasseri	GO:0022857	PTTA – passive transmembrane transporter activity	Adherence, aggregation, and exopolysaccharide synthesis
L. crispatus/L.paragasseri	GO:0016787	Hydrolase transferase	Biosurfactant synthesis
L. crispatus/L.paragasseri	GO:0022857	PTTA – passive transmembrane transporter activity	Adherence, aggregation, and exopolysaccharide synthesis
L. gasseri/L.paragasseri	GO:0006012	GM - Galactose metabolism	Adherence, aggregation, and exopolysaccharide synthesis
L. gasseri/L.paragasseri	GO:0006457	CA - Chaperone activity	Adherence, aggregation, and exopolysaccharide synthesis
L. gasseri/L.paragasseri	GO:0022857	PTTA – passive transmembrane transporter activity	Adherence, aggregation, and exopolysaccharide synthesis

L. gasseri/L.paragasseri	GO:0097367	CDB – Carbohydrate derivative binding	Adherence, aggregation, and exopolysaccharide synthesis
L. gasseri/L.paragasseri	GO:0008324	TTA – Transmembrane transporter activity	Bacteriocin synthesis
L. gasseri/L.paragasseri	GO:0140359	ABC transporter	Bacteriocin synthesis
L. gasseri/L.paragasseri	GO:0016787	Hydrolase transferase	Biosurfactant synthesis
L. gasseri/L.paragasseri	GO:1901135	GCMP – Glycosyl compound metabolic process	Biosurfactant synthesis
L. gasseri/L.paragasseri	GO:0008152	OSMP – Organic substance metabolic process	Hydrogen peroxide and organic acid biosynthesis<br>D/L-lactate metabolic processes
L. gasseri/L.paragasseri/crispatus	GO:0016787	Hydrolase transferase	Biosurfactant synthesis
L. gasseri/L.paragasseri/crispatus	GO:0022857	PTTA – passive transmembrane transporter activity	Adherence, aggregation, and exopolysaccharide synthesis
