# Supplementary material for: Women’s health is a team effort: probiogenomics supports the development of a multi-species vaginal probiotic
Source: Cell Mol Life Sci. 2026 Feb 26;83(1):132. doi: 10.1007/s00018-026-06107-2 (PMC12957687; doi:10.1007/s00018-026-06107-2)
Supplement: Supplementary file 5 — Supplementary Material 5 (PDF 99.5 KB) [file 18_2026_6107_MOESM5_ESM.pdf]

Women's health is a team effort: probiogenomics supports the development of a multi-species vaginal probiotic

Chiara Maria Calvanese[1], Vincenzo Valentino[1], Annachiara De Prisco[2], Serena Allesina[2], Angela Amoruso[2], Francesca Deidda[2], Annalisa Visciglia[2], Danilo Ercolini[1,3], Marco Pane[2], Francesca De Filippis[1,3*]

[1] Department of Agricultural Sciences, University of Naples Federico II, P.zza Carlo di Borbone 1, 80055 Portici (NA), Italy

[2]Probiotical Research S.r.l., via Enrico Mattei 3, 28100 Novara, Italy

[3] Task Force on Microbiome Studies, University of Naples Federico II, Corso Umberto I 43, 80100 Napoli, Italy

Journal: Cellular and Molecular Life Sciences

Corresponding Author:

Prof. Francesca De Filippis

Department of Agricultural Sciences, University of Naples Federico II, Via Università 100, 80055 Portici, Italy

e-mail: francesca.defilippis@unina.it; Phone: +39 081-2539388

ORCID: 0000-0002-3474-2884

Online Resource 5. Contigs in the genomes of newly isolated strains predicted as belonging to plasmids.

**Genome ID**	**Plasmidic Contig ID**
B5_*L.crispatus*	NODE_54_length_12471_cov_1681.597168
C2_*L.crispatus*	NODE_68_length_11124_cov_1744.180423
C3_ *L.crispatus*	NODE_68_length_11124_cov_1494.148643
J1_ *L.paragasseri*	NODE_6_length_26819_cov_3870.155721
ID686_ DSM 32277 _*L.fermentum*	NODE_7_length_18696_cov_137.345
ID1969_ DSM 32405_ *L_gasseri*	MB79-ID1969_contig_8
Q1 *L.crispatus*	NODE_9_length_43888_cov_887.506156
Q2_*L.gasseri*	NODE_15_length_2740_cov_1.284519
T1_*L.crispatus*	NODE_9_length_46309_cov_1141.134811
U2_*L.crispatus*	NODE_4_length_74800_cov_1045.453869
X2_*L.crispatus*	NODE_85_length_6470_cov_2018.200975
Z1_*L.crispatus*	NODE_84_length_6198_cov_1577.094627
